# Supplementary material for: Comparison of the Transcriptomic Signatures in Pediatric and Adult CML
Source: Cancers (Basel). 2021 Dec 14;13(24):6263. doi: 10.3390/cancers13246263 (PMC8699058; doi:10.3390/cancers13246263)
Supplement: Supplementary file 1 [file cancers-13-06263-s001.zip › cancers-1479549-supplementary.pdf]

cancers

Supplementary Materials

Comparison of the Transcriptomic Signatures in Pediatric and Adult CML

Minyoung Youn, Stephanie M. Smith, Alex Gia Lee, Hee-Don Chae, Elizabeth Spiteri, Jason Erdmann, Ilana Galperin, Lara Murphy Jones, Michele Donato, Parveen Abidi, Henrique Bittencourt, Norman Lacayo, Gary Dahl, Catherine Aftandilian, Kara L. Davis, Jairo A. Matthews, Steven M. Kornblau, Min Huang, Nathan Sumarsono, Michele S. Redell, Cecilia H. Fu, I-Ming Chen, Todd A. Alonzo, Elizabeth Eklund, Jason Gotlib, Purvesh Khatri, E. Alejandro Sweet-Cordero, Nobuko Hijiya, and Kathleen M. Sakamoto

Methods S1:

FISH

CML CD34+ cells were isolated by magnetic sorting (Miltenyi Biotec, Inc.: Sunnyvale, CA, USA).Sorted cells underwent hypotonic cell expansion and methanol/acetic acid fixation. Slides were prepared, pretreated with 2xSSC, alcohol dehydrated, and hybridized with BCR-ABL t(9;22) dual color, dual fusion translocation probes (Abbott/Vysis). Analysis of 200 interphase nuclei per slide was performed by a fluorescent microscope. Observed signal patterns were tallied andcategorized, as per clinical standards.

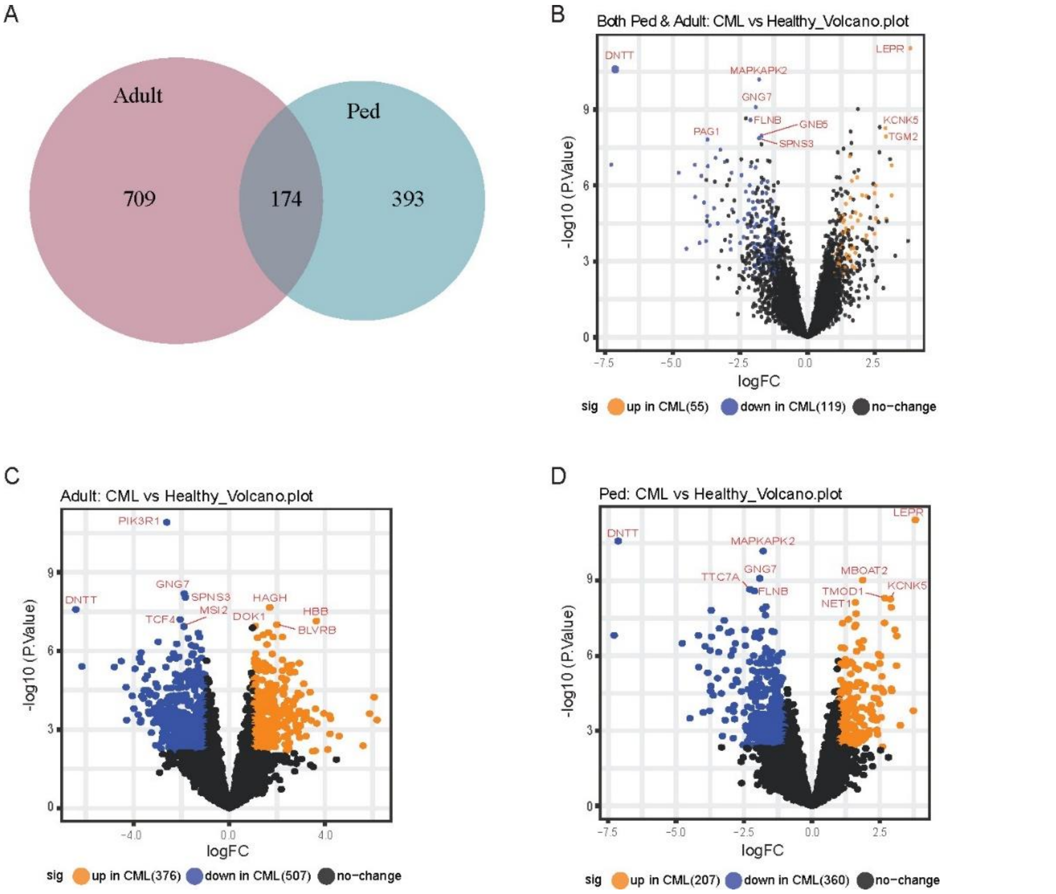

Figure S1. Volcano plots show differentially expressed genes in each comparison. (**A**) Venn diagram with the number of differentially expressed genes (DEG) between CML CD34+ cells and healthy

control CD34+ cells in each age group. DEG defined as FDR of ≤ 0.05 and absolute log2 fold-change > 1. (**B**) Volcano plot with commonly regulated genes in both pediatric and adult CML CD34+ cells. (**C**) Volcano plot with DEG comparing adult CML CD34+ cells vs. adult healthy control CD34+ cells. (**D**) Volcano plot with DEG comparing pediatric CML CD34+ cells vs. pediatric healthy control CD34+ cells.

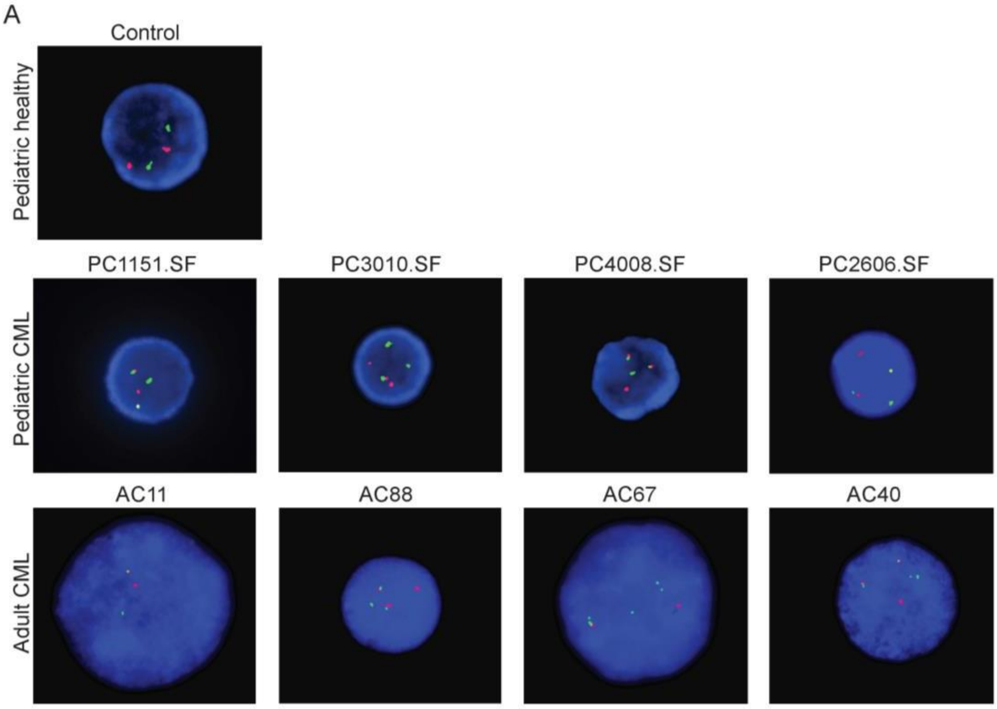

B

Sample IDs	% of BCR-ABL t(9;22) in CD34+ cells (n=200 nuclei)	Variant types
**Pediatric CMLs**		
PC1151.SF	97	double fusion
PC3010.SF	100	Single fusion and single extra for both
PC4008.SF	100	double fusion
PC2606.SF	99	double fusion
**Adult CMLs**		
AC11	99.5	Single fusion
AC88	94	double fusion
AC67	95	Single fusion and an extra BCR
AC40	92	double fusion

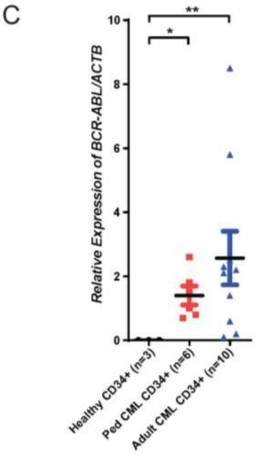

Figure S2. BCR-ABL1 fusions were detected in ≥ 92% of CD34+ cells from both pediatric and adult CML patient samples by FISH analysis. * Green: BCR, Red: ABL1. (**A**) Representative images of

BCR-ABL+ cells in indicated samples. **(B)** Percentages of BCR-ABL+ cells in each CML CD34+ sample. **(C)** BCR-ABL expression was detected by RT-qPCR in CD34+ cells from pediatric and adult patients with CML but not in CD34+ cells from healthy donors.

A

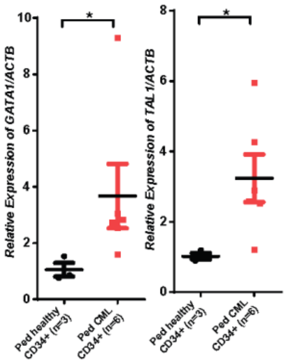

B

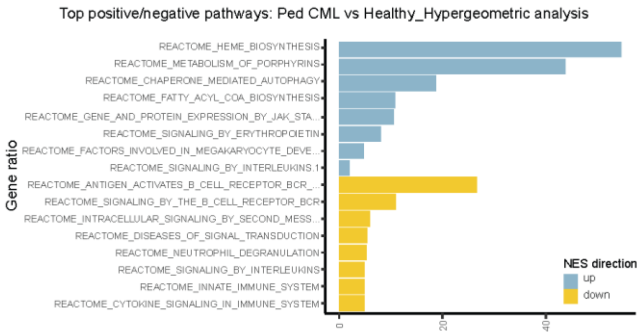

C

Pathway	GeneRatio	p value	group
REACTOME_RUNX3_REGULATES_NOTCH_SIGNALING	21.43	1.56E-03	down
REACTOME_NOTCH4_INTRACELLULAR_DOMAIN_REGULATES_TRANSCRIPTION	15.00	4.54E-03	down
REACTOME_NOTCH_HLH_TRANSCRIPTION_PATHWAY	14.29	1.24E-03	down
REACTOME_NOTCH1_INTRACELLULAR_DOMAIN_REGULATES_TRANSCRIPTION	12.77	1.42E-04	down
REACTOME_NOTCH3_INTRACELLULAR_DOMAIN_REGULATES_TRANSCRIPTION	12.00	8.61E-03	down
REACTOME_SIGNALING_BY_NOTCH1	8.22	1.55E-03	down
REACTOME_SIGNALING_BY_NOTCH4	7.32	2.81E-03	down
REACTOME_SIGNALING_BY_NOTCH1_IN_CANCER	6.90	1.72E-02	down
REACTOME_SIGNALING_BY_NOTCH	3.77	2.21E-02	down
REACTOME_REPRESSION_OF_WNT_TARGET_GENES	21.43	1.56E-03	down
REACTOME_BETA_CATENIN_INDEPENDENT_WNT_SIGNALING	4.83	1.24E-02	down
REACTOME_TCF_DEPENDENT_SIGNALING_IN_RESPONSE_TO_WNT	3.88	1.87E-02	down
REACTOME_SIGNALING_BY_WNT	3.67	1.09E-02	down
REACTOME_REGULATION_OF_SIGNALING_BY_CBL	22.73	2.93E-05	down

D

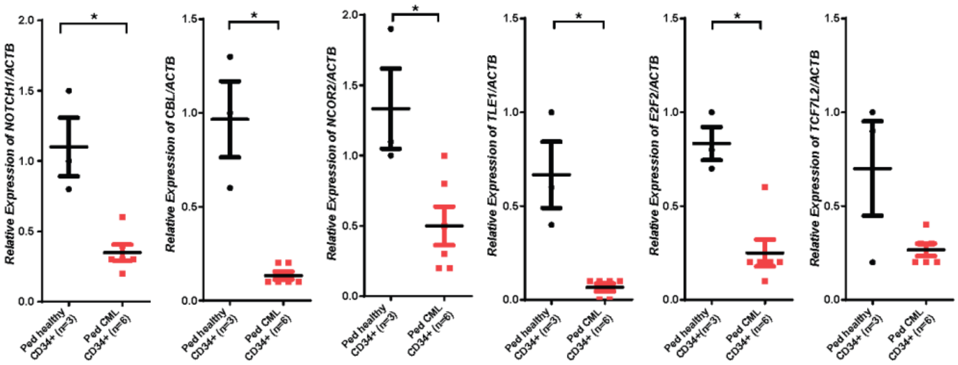

Figure S3. Differentially expressed genes and pathways comparing pediatric CML CD34+ cells vs. pediatric healthy control CD34+ cells. (**A**) GATA1 and TAL1 expression were significantly increased in pediatric CML CD34+ cells compared to pediatric healthy CD34+ cells. (**B**) Barplot shows top REACTOME pathways in pediatric CML. CD34+ cells compared to pediatric healthy CD34+ cells. (**C**) NOTCH, WNT, and CBL pathways were significantly downregulated in pediatric CML compared to pediatric healthy controls. (**D**) Several genes involved in NOTCH, WNT, and CBL pathways demonstrate decreased expressions in pediatric CML CD34+ cells compared to pediatric healthy CD34+ cells. Each spot on the graph represents an individual sample. mRNA levels were assessed by RT-qPCR and normalized against *beta-actin (ACTB)* expression. * $p < 0.05$.

A

Ped healthy vs Adult healthy_DEG list

gene	logFC	P.Value	adj.P.Val	gene	logFC	P.Value	adj.P.Val
LYZ	2.19	0.018	0.783	DNAJC2	-1.00	0.017	0.783
NLRP3	1.53	0.041	0.783	NAA15	-1.00	0.007	0.783
IGF2R	1.52	0.009	0.783	ANKRD49	-1.01	0.040	0.783
PLK2	1.46	0.033	0.783	UPF3B	-1.01	0.030	0.783
BHLHE40	1.39	0.017	0.783	GKAP1	-1.02	0.007	0.783
SAMHD1	1.38	0.036	0.783	RICTOR	-1.02	0.004	0.783
C10orf54	1.35	0.035	0.783	BEX2	-1.04	0.010	0.783
CXCR4	1.35	0.009	0.783	DCTN6	-1.04	0.005	0.783
IRS2	1.35	0.009	0.783	PSMA1	-1.05	0.011	0.783
ITGB2	1.35	0.012	0.783	RSBN1	-1.05	0.004	0.783
ITPRIP	1.32	0.003	0.783	MX1	-1.07	0.027	0.783
SRGN	1.19	0.023	0.783	PHIP	-1.07	0.024	0.783
CPVL	1.18	0.030	0.783	SOX4	-1.07	0.020	0.783
FKBP5	1.18	0.005	0.783	WDR60	-1.08	0.012	0.783
P2RY2	1.17	0.028	0.783	RBM25	-1.09	0.039	0.783
C3orf58	1.15	0.038	0.783	CHRM3	-1.10	0.014	0.783
PITPNM1	1.15	0.024	0.783	CNPY2	-1.10	0.011	0.783
COTL1	1.15	0.045	0.799	KIAA0907	-1.10	0.006	0.783
GRN	1.14	0.033	0.783	TFPI	-1.10	0.001	0.783
TNFRSF10D	1.12	0.009	0.783	YIPF4	-1.10	0.001	0.783
CD22	1.07	0.007	0.783	KRT8	-1.10	0.044	0.796
PIK3AP1	1.07	0.003	0.783	CEP290	-1.11	0.028	0.783
PSAP	1.07	0.036	0.783	NEMF	-1.12	0.028	0.783
NAGA	1.06	0.014	0.783	ZNF493	-1.12	0.012	0.783
FAM107B	1.05	0.015	0.783	PNN	-1.13	0.022	0.783
IL16	1.05	0.007	0.783	NHLRC3	-1.14	0.010	0.783
PDE4B	1.01	0.018	0.783	HSPE1	-1.15	0.010	0.783
				SMIM24	-1.15	0.035	0.783
				ZRANB2	-1.15	0.001	0.783
				ESF1	-1.16	0.007	0.783
				MYCT1	-1.16	0.017	0.783
				RPS6KA5	-1.17	0.003	0.783
				CWF19L2	-1.18	0.008	0.783
				SLF1	-1.18	0.003	0.783
				FAM133B	-1.19	0.026	0.783
				ZMAT1	-1.20	0.029	0.783
				PGM2L1	-1.21	0.042	0.783
				SRSF11	-1.21	0.028	0.783
				BEST1	-1.22	0.013	0.783
				SLC4A7	-1.22	0.009	0.783
				ZBTB8A	-1.22	0.006	0.783
				CSPP1	-1.23	0.006	0.783
				PLCB4	-1.26	0.023	0.783
				HTR1F	-1.35	0.033	0.783
				THRB	-1.43	0.026	0.783
				RPL36A	-1.44	0.028	0.783
				MACC1	-1.49	0.021	0.783
				SH3D19	-1.49	0.001	0.783
				MAP9	-1.60	0.000	0.783
				LUC7L3	-1.63	0.005	0.783
				ANKRD36C	-1.73	0.010	0.783
				ANKRD36	-1.89	0.006	0.783
				IFI44L	-1.97	0.009	0.783

B

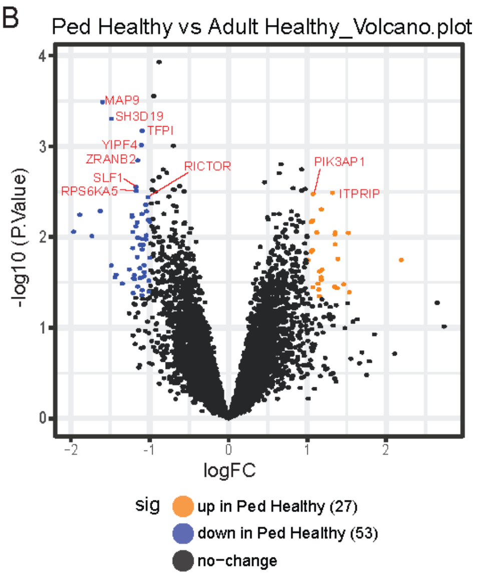

Figure S4. Comparison of pediatric healthy and adult healthy CD34+ cells does not show significant differences. (**A**) Table and (**B**) Volcano plot show DEG in pediatricand adult healthy controls. DEG defined as FDR of ≤0.8 and absolute log2 fold-change >1.

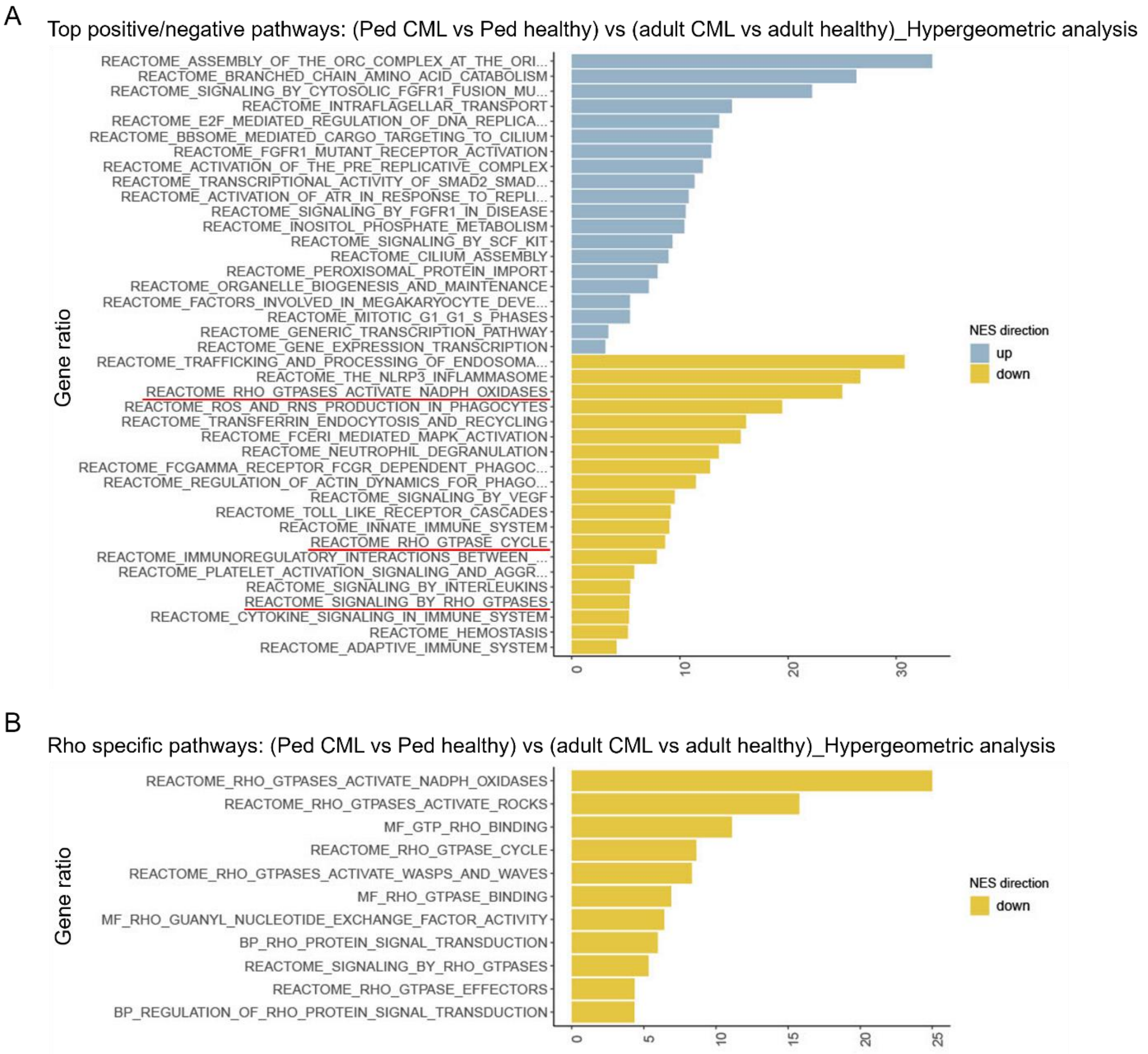

Figure S5. Differentially expressed pathways for (pediatric CML vs pediatric healthy) vs (adult CML vs adult healthy). (**A**) top REACTOME pathways (**B**) Rho pathways only; MF and BP are GP terms for Molecular Function and Biological Process, respectively.

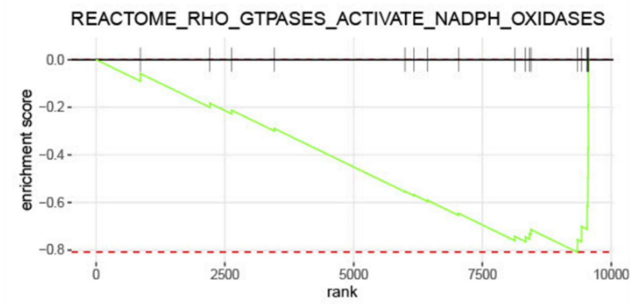

Pathway	pval	padj	ES	NES
REACTOME_RHO_GTPASES_ACTIVATE_NADPH_OXIDASES	0.0025	0.0290	-0.8088	-2.3838

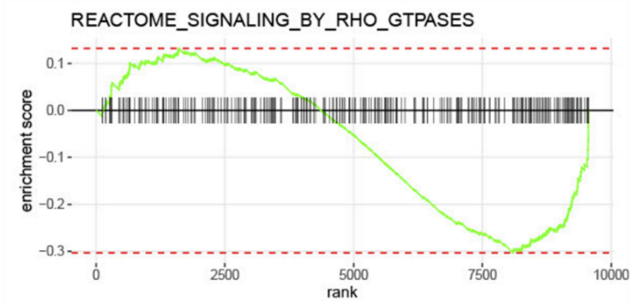

Pathway	pval	padj	ES	NES
REACTOME_SIGNALING_BY_RHO_GTPASES	0.0065	0.0370	-0.3035	-1.4508

Figure S6. GSEA-enrichment plots of Rho pathways between (pediatric CML vs pediatric healthy) vs (adult CML vs adult healthy).

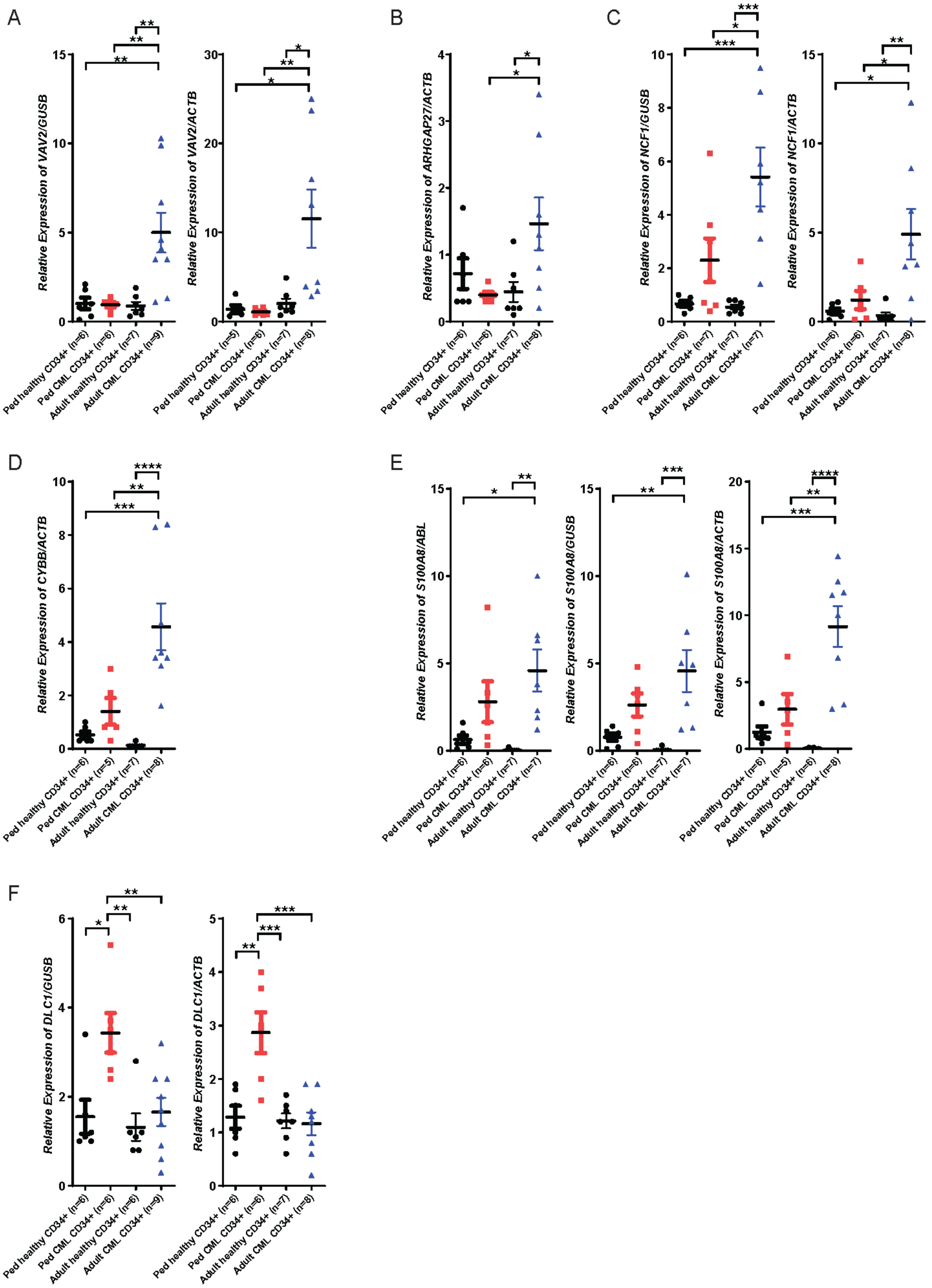

Figure S7. Several genes involved in the Rho pathway were differentially expressed between pediatric CML CD34+ cells and adult CML CD34+ cells. (**A–F**) mRNA expression levels of dysregulated genes were assessed by RT-qPCT and normalized against *Abelson* (*ABL*), *beta-glucuronidase* (*GUSB*), or *beta-actin* (*ACTB*) expression. Data were graphed as mean ± SEM. Each spot on the graph represents an individual sample. p values forstatistical significance were obtained using unpaired Student t-test or ANOVA test (Tukey's multiple comparison). * $p < 0.05$, ** $p < 0.01$, *** $p < 0.001$, **** $p < 0.0001$.

Table S1. Demographic and clinical characteristics of CML patients andhealthy controls.

Sample ID	Sex	Age	Race/Ethnicity	CML Phase	WBC Count	Platelet Count	Spleen Size
**Pediatric CML patients**							
PC1151.SF	M	13	Hispanic	Chronic	291	626	8
PC3010.SF	M	14	Asian	Chronic	351	617	5
PC2606.SF	M	9	Other	Chronic	255	627	2
PC4008.SF	M	17	Asian	Chronic	488	439	5
PC5907.CG	M	10	Asian	Chronic	108	698	Unknown
PC6128.CG	M	10	Asian	Chronic	95	1047	Unknown
PC8267.CG	F	11	Hispanic	Unknown	Unknown	Unknown	Unknown
PC9495.CG	M	9	White	Unknown	Unknown	Unknown	Unknown
PC2300.SF	M	5	White	Chronic	57	870	0
**Adult CML patients**							
AC10.SF	M	33	Asian	Chronic	370	253	17
AC33.SF	M	55	White	Chronic	57	348	0
AC36.SF	M	62	White	Chronic	37	590	0
AC48.SF	M	23	Hispanic	Chronic	260	134	4
AC64.SF	F	30	Hispanic	Chronic	273	262	0
AC40	M	68	White	Chronic	64	355	0
AC82	M	62	White	Chronic	136	456	0
AC11	M	44	Black	Chronic	167	205	0
AC88	M	62	White	Chronic	126	187	0
AC67	M	53	White	Chronic	149	371	0
**Pediatric healthy controls**							
PN71.UM	F	12	Asian				
PN81.UM	M	10	White				
PN12	M	19	Other				
PN14	M	19	Other				
PN31	F	19	Hispanic				
PN46	M	19	Black				
PN85	F	18	Black				
PN06	M	19	Black				
PN74	F	19	White				
PN18	M	19	Black				
**Adult healthy controls**							
AN96	M	33	Black				
AN69	M	35	Hispanic				
AN173	M	31	Black				
AN369	F	32	Black				
AN52	M	29	Black				
AN53	M	44	Other				
AN55	M	28	Black				
AN35.LZ	F	24	Unknown				
AN73.LZ	M	25	Black				
AN62.LZ	M	21	Black				

M=male; F=female; Age is reported in years; Spleen size is reported in centimetersbelow the costal margin.

Table S2. All DEG in pediatric CML compared to adult CML, sorted according to logFC level. *p* values were obtained using hypergeometric test.

Upregulated in Pediatric CML compared to Adult CML				Downregulated in Pediatric CML compared to Adult CML			
**gene**	**logFC**	***p*.Value**	**adj.*p*.Val**	**Gene**	**logFC**	***p*.Value**	**adj.*p*.Val**
PLOD2	3.62	5.00E-04	1.67E-02	GRINA	-1.51	2.00E-04	1.33E-02
FAM199X	3.52	0.00E+00	9.20E-03	NUDT16	-1.52	1.00E-03	2.18E-02
BEND4	3.52	1.00E-04	1.18E-02	C16orf74	-1.54	1.30E-03	2.32E-02
CD40LG	3.41	1.00E-04	1.17E-02	FAM50A	-1.55	3.00E-04	1.40E-02
PKIA	3.32	0.00E+00	1.05E-02	IL18BP	-1.56	2.50E-03	3.10E-02
OXR1	3.28	1.00E-04	1.11E-02	CARD16	-1.57	2.30E-03	3.02E-02
PEX7	3.27	1.00E-04	1.18E-02	ZFHX3	-1.57	4.00E-03	3.79E-02
NUDCD1	3.17	9.00E-04	2.08E-02	HOOK2	-1.58	0.00E+00	9.70E-03
SGPP1	3.15	1.00E-04	1.22E-02	ADGRE5	-1.59	7.00E-04	1.88E-02
NEMP2	3.14	1.00E-04	1.22E-02	S100A4	-1.6	3.20E-03	3.38E-02
MYEF2	3.13	0.00E+00	1.02E-02	JSRP1	-1.61	1.00E-04	1.19E-02
TADA1	3.11	0.00E+00	9.40E-03	PLEKHO1	-1.61	3.00E-04	1.47E-02
WDR44	3.09	0.00E+00	9.70E-03	IL10RB	-1.61	4.00E-04	1.67E-02
FBLN2	3.09	3.00E-04	1.47E-02	HSPA1A	-1.61	3.10E-03	3.35E-02
BMI1	3.08	3.00E-04	1.52E-02	CACFD1	-1.62	5.00E-04	1.67E-02
VANGL2	3.08	6.00E-04	1.81E-02	TSPO	-1.63	1.00E-04	1.18E-02
SH3BGRL2	3.07	3.00E-04	1.42E-02	LST1	-1.63	2.50E-03	3.13E-02
WFDC1	3.04	4.00E-04	1.58E-02	SAT2	-1.64	6.00E-04	1.82E-02
MYCT1	3.02	4.00E-04	1.64E-02	CYBA	-1.65	1.00E-04	1.22E-02
LYSMD3	3.01	1.00E-04	1.22E-02	APOL1	-1.65	7.00E-04	1.91E-02
C5	2.9	0.00E+00	9.90E-03	HIST1H2BG	-1.65	2.70E-03	3.18E-02
MBTPS2	2.9	3.00E-04	1.42E-02	LTB4R	-1.66	1.60E-03	2.61E-02
ZNF675	2.88	1.00E-04	1.22E-02	S100A6	-1.66	4.20E-03	3.86E-02
FNBP1L	2.87	2.00E-04	1.29E-02	BRI3	-1.67	1.20E-03	2.29E-02
GALNT12	2.86	4.00E-04	1.67E-02	RXRA	-1.68	7.00E-04	1.90E-02
FAM160B1	2.85	7.00E-04	1.91E-02	SULT1A1	-1.68	1.20E-03	2.31E-02
TTK	2.85	1.20E-03	2.29E-02	TCIRG1	-1.7	2.00E-03	2.85E-02
ZSCAN12	2.82	2.00E-04	1.33E-02	TRPM2	-1.7	2.00E-03	2.88E-02
MYCN	2.82	1.50E-03	2.50E-02	IL6R	-1.71	1.40E-03	2.45E-02
CLDN10	2.79	1.20E-03	2.31E-02	HLA.DMB	-1.74	4.00E-04	1.63E-02
SCOC	2.77	1.00E-04	1.22E-02	AHNAK	-1.74	1.60E-03	2.63E-02
FANCF	2.77	1.10E-03	2.25E-02	IER2	-1.74	3.60E-03	3.58E-02
NAA30	2.76	1.00E-04	1.22E-02	CD72	-1.74	3.60E-03	3.59E-02
ZNF776	2.74	2.00E-04	1.33E-02	PTPRE	-1.74	6.00E-03	4.70E-02
FKBP14	2.74	4.00E-04	1.58E-02	OPRL1	-1.74	6.50E-03	4.88E-02
CEP70	2.71	1.00E-04	1.18E-02	ANKRD11	-1.75	3.00E-03	3.32E-02
ZNF518B	2.71	2.00E-04	1.33E-02	HLA.F	-1.76	5.00E-03	4.22E-02
EXTL2	2.71	5.00E-04	1.76E-02	UNC93B1	-1.77	4.00E-04	1.63E-02
RRM2B	2.7	1.00E-03	2.21E-02	ANXA2	-1.77	3.40E-03	3.49E-02
SOCS6	2.67	1.00E-04	1.22E-02	MYO1F	-1.78	4.00E-04	1.58E-02
DNAJC18	2.67	2.00E-04	1.23E-02	MVP	-1.78	6.00E-04	1.80E-02
MIER3	2.65	2.00E-04	1.33E-02	ZDHHC1	-1.79	5.00E-04	1.67E-02
IGF2BP2	2.65	5.90E-03	4.67E-02	SH2B2	-1.79	3.30E-03	3.45E-02
CEP83	2.61	2.00E-04	1.32E-02	CTSD	-1.81	6.00E-04	1.84E-02
YOD1	2.61	3.00E-04	1.40E-02	LGALS1	-1.81	2.10E-03	2.91E-02
HOXB2	2.61	7.00E-04	1.91E-02	DUSP2	-1.81	2.80E-03	3.21E-02
C12orf66	2.6	6.00E-04	1.86E-02	TNFAIP8L2	-1.83	4.00E-04	1.64E-02
XPR1	2.59	3.00E-04	1.40E-02	KLF6	-1.83	7.00E-04	1.91E-02
SLC24A3	2.59	1.50E-03	2.53E-02	GRN	-1.83	1.20E-03	2.31E-02

DIRAS1	2.58	2.10E-03	2.92E-02	AGTRAP	-1.84	1.00E-04	1.22E-02
GSTCD	2.57	3.00E-04	1.52E-02	TLE4	-1.84	2.00E-04	1.40E-02
HSPA13	2.57	2.70E-03	3.17E-02	IRF7	-1.85	1.60E-03	2.61E-02
ZBTB11	2.55	7.00E-04	1.91E-02	RRBP1	-1.85	2.40E-03	3.09E-02
PACRGL	2.54	4.00E-04	1.63E-02	CAMK1	-1.86	4.00E-04	1.58E-02
ZNF136	2.54	7.00E-04	1.89E-02	C4orf48	-1.87	7.00E-04	1.91E-02
AIF1L	2.54	5.20E-03	4.31E-02	BEST1	-1.88	5.00E-04	1.77E-02
SERAC1	2.52	0.00E+00	9.90E-03	C1orf162	-1.91	3.00E-03	3.34E-02
RNF217	2.52	1.10E-03	2.26E-02	SH3TC1	-1.91	6.80E-03	4.94E-02
ATP8A1	2.52	5.40E-03	4.40E-02	RRAS	-1.92	3.20E-03	3.38E-02
USP46	2.49	1.00E-04	1.22E-02	HCST	-1.93	2.00E-04	1.38E-02
ARHGAP18	2.49	3.00E-04	1.40E-02	DUSP1	-1.93	2.80E-03	3.21E-02
LATS1	2.49	1.40E-03	2.49E-02	NFKB2	-1.94	1.00E-03	2.21E-02
TMEM231	2.48	7.00E-04	1.91E-02	HRH2	-1.94	2.40E-03	3.10E-02
LRRC37B	2.48	1.10E-03	2.26E-02	FOS	-1.94	4.20E-03	3.87E-02
NEK7	2.46	8.00E-04	2.05E-02	CCDC88B	-1.96	1.20E-03	2.29E-02
SEC23A	2.46	9.00E-04	2.11E-02	CECR1	-1.96	6.40E-03	4.78E-02
SVOPL	2.45	4.00E-04	1.63E-02	NINJ1	-1.96	6.60E-03	4.88E-02
PLEKHA4	2.44	1.40E-03	2.46E-02	ITGAL	-1.97	1.00E-04	1.18E-02
ZNF569	2.43	1.00E-04	1.22E-02	SRGAP2C	-1.97	6.40E-03	4.78E-02
ZBTB34	2.43	6.00E-04	1.86E-02	CYTH4	-1.98	6.00E-04	1.79E-02
ZNF549	2.43	1.80E-03	2.75E-02	ISG15	-1.99	2.50E-03	3.10E-02
RCN2	2.42	1.80E-03	2.75E-02	PSTPIP1	-1.99	3.30E-03	3.45E-02
CHML	2.41	1.40E-03	2.45E-02	METRNL	-1.99	5.40E-03	4.38E-02
HOXA5	2.41	3.60E-03	3.57E-02	LSP1	-2.01	1.00E-04	1.18E-02
WDR35	2.4	3.00E-04	1.52E-02	SLC16A3	-2.01	6.00E-04	1.82E-02
MTHFD2L	2.39	1.20E-03	2.31E-02	SLC43A2	-2.01	2.00E-03	2.84E-02
KLHL28	2.38	2.00E-04	1.38E-02	CST3	-2.02	2.00E-04	1.31E-02
DCUN1D1	2.38	5.00E-04	1.67E-02	TNFAIP2	-2.03	3.10E-03	3.38E-02
ZNF736	2.38	7.00E-04	1.91E-02	CEBPB	-2.07	1.60E-03	2.63E-02
ZBTB38	2.36	4.30E-03	3.88E-02	CDC42EP2	-2.09	2.00E-04	1.27E-02
METTL25	2.35	3.70E-03	3.61E-02	TNFSF10	-2.09	1.20E-03	2.29E-02
CCSAP	2.34	4.00E-04	1.63E-02	SPON2	-2.1	6.00E-04	1.85E-02
TSPYL5	2.34	4.40E-03	3.91E-02	BTG2	-2.1	7.00E-04	1.91E-02
FREM1	2.33	1.80E-03	2.72E-02	CKAP4	-2.1	4.10E-03	3.84E-02
ZNF329	2.32	9.00E-04	2.07E-02	C10orf54	-2.11	1.80E-03	2.75E-02
SWT1	2.32	1.50E-03	2.51E-02	RHOB	-2.12	1.80E-03	2.75E-02
BRIP1	2.32	2.60E-03	3.17E-02	ZFP36	-2.12	4.60E-03	4.02E-02
SLAIN1	2.3	1.00E-03	2.22E-02	IGHM	-2.13	8.00E-04	1.98E-02
TMX3	2.3	1.70E-03	2.68E-02	CD79A	-2.13	3.50E-03	3.55E-02
PBX1	2.29	1.10E-03	2.28E-02	RGS2	-2.16	4.00E-03	3.76E-02
SLCO4A1	2.26	1.10E-03	2.25E-02	IL17RA	-2.19	2.00E-04	1.31E-02
ZNF614	2.26	1.40E-03	2.46E-02	ARL4C	-2.19	1.20E-03	2.30E-02
ACBD5	2.25	1.20E-03	2.31E-02	MSRB1	-2.22	3.00E-04	1.42E-02
THAP1	2.23	4.00E-04	1.58E-02	NKG7	-2.24	2.90E-03	3.25E-02
MFN1	2.23	1.10E-03	2.24E-02	ID2	-2.24	4.50E-03	4.00E-02
GTPBP8	2.22	4.00E-04	1.56E-02	RHBDF2	-2.27	7.00E-04	1.89E-02
DMXL1	2.21	2.70E-03	3.17E-02	CYSTM1	-2.27	2.20E-03	2.97E-02
TWF1	2.2	3.30E-03	3.42E-02	B3GALT4	-2.29	1.10E-03	2.26E-02
SMO	2.2	6.10E-03	4.73E-02	CEBPD	-2.3	2.70E-03	3.20E-02
SCML2	2.19	1.70E-03	2.66E-02	GBP2	-2.32	1.20E-03	2.31E-02
FAM234B	2.19	6.20E-03	4.77E-02	FCER1G	-2.33	6.40E-03	4.81E-02
FAM19A2	2.19	6.80E-03	4.96E-02	FYB	-2.34	5.00E-04	1.71E-02
TBC1D16	2.18	4.70E-03	4.07E-02	ADAP1	-2.37	1.20E-03	2.31E-02

BBS7	2.17	6.00E-04	1.86E-02	CFP	-2.38	1.20E-03	2.30E-02
RAB39B	2.16	3.80E-03	3.65E-02	HVCN1	-2.4	2.50E-03	3.14E-02
CEP162	2.16	4.10E-03	3.79E-02	NAGK	-2.44	0.00E+00	7.10E-03
CGRRF1	2.16	4.60E-03	4.02E-02	IFI27L2	-2.45	2.00E-04	1.27E-02
LEPR	2.15	0.00E+00	1.00E-04	JUN	-2.46	4.00E-04	1.56E-02
KCNQ5	2.15	2.70E-03	3.20E-02	P2RX7	-2.48	2.00E-04	1.23E-02
TRIB2	2.14	2.00E-03	2.84E-02	PLAUR	-2.49	6.30E-03	4.78E-02
RFX7	2.14	6.70E-03	4.91E-02	PLK3	-2.5	2.00E-04	1.23E-02
CCDC171	2.13	4.40E-03	3.91E-02	TYROBP	-2.52	4.50E-03	4.00E-02
DTD2	2.12	5.00E-03	4.23E-02	TNFRSF1B	-2.53	5.00E-04	1.78E-02
THAP5	2.12	5.90E-03	4.67E-02	GRK5	-2.53	6.00E-04	1.84E-02
EOGT	2.11	4.00E-04	1.63E-02	APOBR	-2.62	2.00E-04	1.29E-02
COG6	2.11	4.40E-03	3.91E-02	DOK2	-2.63	6.00E-04	1.80E-02
SPRY2	2.09	3.00E-04	1.47E-02	SLC2A6	-2.64	1.40E-03	2.47E-02
ARL5B	2.09	2.80E-03	3.25E-02	EGR1	-2.64	1.70E-03	2.72E-02
FZD1	2.09	4.50E-03	4.00E-02	JAML	-2.64	2.00E-03	2.85E-02
ZNF367	2.08	3.40E-03	3.45E-02	S100A11	-2.72	8.00E-04	1.91E-02
KCTD9	2.07	1.80E-03	2.72E-02	FGR	-2.8	3.20E-03	3.38E-02
PAQR3	2.06	1.60E-03	2.61E-02	CTSH	-2.82	1.10E-03	2.25E-02
MED21	2.06	2.60E-03	3.15E-02	NBPF26	-2.82	5.40E-03	4.41E-02
AASDH	2.05	2.50E-03	3.13E-02	LTB	-2.84	1.00E-03	2.22E-02
C2orf69	2.04	1.90E-03	2.79E-02	HCK	-2.85	6.00E-04	1.86E-02
STXBP1	2.04	2.40E-03	3.10E-02	CYBB	-2.87	5.10E-03	4.26E-02
IL6ST	2.04	3.40E-03	3.46E-02	SLC11A1	-2.9	3.00E-04	1.45E-02
PLEKHA8	2.03	3.50E-03	3.52E-02	ASGR1	-2.93	2.00E-04	1.23E-02
PDZD8	2.02	0.00E+00	7.10E-03	LILRB2	-2.96	3.60E-03	3.56E-02
MTRF1L	2.02	2.80E-03	3.25E-02	POU2F2	-3	1.00E-04	1.22E-02
SNUPN	2.02	4.20E-03	3.87E-02	KLF2	-3.05	2.00E-04	1.29E-02
ACTR6	2.01	1.10E-03	2.25E-02	NCF1	-3.31	6.20E-03	4.75E-02
SCYL2	2.01	1.50E-03	2.50E-02	FAM20C	-3.42	7.00E-04	1.88E-02
ZNF761	2.01	4.20E-03	3.86E-02	PLEC	-3.63	2.00E-04	1.23E-02
RALGAPA1	2.01	5.90E-03	4.66E-02	TYMP	-3.96	3.00E-04	1.47E-02
NEDD4	2	1.50E-03	2.56E-02	FCN1	-4.17	6.00E-04	1.80E-02
OMA1	1.99	7.00E-04	1.91E-02	S100A9	-4.3	1.10E-03	2.24E-02
VCPKMT	1.99	1.10E-03	2.26E-02	S100A8	-4.36	1.10E-03	2.28E-02
CLIC4	1.99	3.10E-03	3.35E-02	SERPINA1	-4.48	1.10E-03	2.26E-02
FKTN	1.99	6.00E-03	4.72E-02	LRP1	-4.85	4.20E-03	3.86E-02
ASAP2	1.98	4.70E-03	4.04E-02				
CFAP97	1.97	1.00E-04	1.18E-02				
BCKDHB	1.97	9.00E-04	2.07E-02				
FGGY	1.97	1.30E-03	2.36E-02				
GAN	1.97	2.90E-03	3.28E-02				
MPP6	1.97	5.60E-03	4.53E-02				
C9orf85	1.96	2.60E-03	3.14E-02				
ZNF880	1.96	3.10E-03	3.36E-02				
PANK3	1.95	3.30E-03	3.42E-02				
PDGFC	1.95	4.00E-03	3.79E-02				
PTBP2	1.95	6.80E-03	4.94E-02				
ABCC4	1.94	0.00E+00	7.10E-03				
GNA13	1.94	1.60E-03	2.61E-02				
TMEM237	1.94	2.40E-03	3.10E-02				
CLC	1.94	2.70E-03	3.19E-02				
PRPF18	1.94	3.10E-03	3.35E-02				
SLC22A5	1.93	2.40E-03	3.10E-02				

ZNF521	1.93	2.40E-03	3.10E-02
RBBP9	1.93	5.50E-03	4.47E-02
SLC35D1	1.92	1.10E-03	2.24E-02
GABPA	1.92	2.20E-03	3.01E-02
FAM83D	1.92	2.90E-03	3.29E-02
SLC41A1	1.92	4.30E-03	3.88E-02
DEPDC1B	1.92	4.70E-03	4.07E-02
ZNF256	1.92	6.80E-03	4.95E-02
TMEM64	1.91	1.00E-04	1.08E-02
RBM45	1.91	2.30E-03	3.03E-02
METTL10	1.91	2.90E-03	3.27E-02
TRIM59	1.9	6.00E-04	1.83E-02
NR1D2	1.9	2.10E-03	2.92E-02
ZNF354A	1.9	3.40E-03	3.45E-02
TRUB1	1.89	1.00E-03	2.21E-02
PCYT1B	1.89	6.10E-03	4.75E-02
ZNF850	1.88	2.50E-03	3.14E-02
SLC25A15	1.86	9.00E-04	2.11E-02
CEP97	1.85	1.20E-03	2.29E-02
SCRN1	1.85	1.30E-03	2.33E-02
MBNL2	1.85	1.70E-03	2.68E-02
ZNF124	1.85	3.20E-03	3.38E-02
DCAF17	1.85	3.70E-03	3.62E-02
NUP54	1.84	2.60E-03	3.14E-02
CYYR1	1.83	2.00E-04	1.33E-02
C1orf109	1.83	7.00E-04	1.89E-02
RHOBTB1	1.82	1.70E-03	2.68E-02
UACA	1.82	2.10E-03	2.93E-02
ZNF711	1.82	2.30E-03	3.07E-02
TMEM106B	1.82	6.70E-03	4.94E-02
ZNF493	1.8	2.80E-03	3.21E-02
MED12L	1.8	3.60E-03	3.59E-02
ZNF717	1.8	5.00E-03	4.22E-02
IFT81	1.8	6.90E-03	4.98E-02
DLC1	1.79	1.00E-04	1.22E-02
TPD52	1.79	2.00E-03	2.86E-02
ZSCAN9	1.78	3.80E-03	3.67E-02
FRS2	1.78	5.60E-03	4.51E-02
KPNA5	1.78	6.60E-03	4.90E-02
RHOBTB3	1.77	1.70E-03	2.66E-02
ZNF726	1.77	5.60E-03	4.51E-02
ZNF568	1.77	5.70E-03	4.55E-02
FNDC3A	1.77	6.10E-03	4.73E-02
SUCLG2	1.76	4.30E-03	3.88E-02
METTL21B	1.74	9.00E-04	2.11E-02
ZNF792	1.74	1.90E-03	2.79E-02
ZNF254	1.73	3.80E-03	3.64E-02
TTC26	1.72	2.90E-03	3.29E-02
MBIP	1.71	1.50E-03	2.50E-02
ACADSB	1.71	1.70E-03	2.69E-02
RRAGB	1.71	2.00E-03	2.88E-02
CAMKMT	1.71	2.70E-03	3.17E-02
POT1	1.71	3.10E-03	3.35E-02
BDH2	1.7	1.50E-03	2.52E-02

LRRC40	1.7	2.70E-03	3.18E-02
PEX1	1.7	4.20E-03	3.86E-02
GPHN	1.7	4.30E-03	3.89E-02
UBE2E3	1.69	1.00E-03	2.21E-02
CDC23	1.69	3.20E-03	3.38E-02
CCNT1	1.69	4.00E-03	3.78E-02
PRKACB	1.68	1.00E-04	1.22E-02
PARPBP	1.68	2.10E-03	2.92E-02
CKAP2	1.66	1.00E-04	1.23E-02
SPAST	1.66	5.00E-04	1.71E-02
THAP12	1.66	3.70E-03	3.61E-02
AHCYL2	1.66	4.60E-03	4.02E-02
MORC3	1.65	2.70E-03	3.17E-02
RIMKLB	1.65	6.70E-03	4.94E-02
DPY19L3	1.64	3.20E-03	3.38E-02
DNAJC6	1.64	4.50E-03	3.97E-02
PIKFYVE	1.63	3.80E-03	3.64E-02
FAM8A1	1.62	3.90E-03	3.71E-02
STK3	1.62	4.20E-03	3.86E-02
CDC14A	1.62	5.40E-03	4.38E-02
TOM1L1	1.62	5.80E-03	4.58E-02
MBNL3	1.61	6.00E-04	1.86E-02
ZNF141	1.6	9.00E-04	2.11E-02
SH3YL1	1.6	4.50E-03	3.99E-02
ZNF664	1.59	0.00E+00	8.50E-03
FBXO28	1.59	4.20E-03	3.86E-02
TWSG1	1.58	2.00E-04	1.38E-02
POLE2	1.58	4.90E-03	4.17E-02
LCLAT1	1.58	5.80E-03	4.61E-02
SSX2IP	1.56	3.50E-03	3.52E-02
TMTC4	1.56	5.60E-03	4.51E-02
PRRC1	1.56	6.50E-03	4.83E-02
LYPLA1	1.55	1.10E-03	2.24E-02
ENOX2	1.55	2.90E-03	3.25E-02
PDK1	1.55	3.20E-03	3.38E-02
STXBP5	1.54	0.00E+00	7.10E-03
PRKCI	1.54	5.00E-04	1.67E-02
ATP13A3	1.54	1.10E-03	2.25E-02
VPS13A	1.54	3.70E-03	3.62E-02
VAV3	1.54	4.00E-03	3.76E-02
DUS4L	1.53	3.80E-03	3.66E-02
CUL4B	1.53	5.50E-03	4.47E-02
ZNF383	1.52	3.90E-03	3.70E-02
PRKAR2B	1.51	0.00E+00	2.10E-03
KIF2A	1.5	4.00E-04	1.63E-02
DNAJC12	1.5	2.60E-03	3.17E-02
PARP11	1.5	5.00E-03	4.21E-02

Table S3. All pathways differentially regulated in pediatric CML comparedto adult CML, sorted according to GeneRatio level. *p* values were obtained using hypergeometric test.

Pathway.	GeneRatio	*p* Value	Group
REACTOME_CROSS_PRESENTATION_OF_PARTICULATE_EXOGENOUS_ANTIGENS_PHAGOSOMES	37.50	1.67E-05	down
REACTOME_METAL_SEQUESTRATION_BY_ANTIMICROBIAL_PROTEINS	33.33	6.73E-04	down
REACTOME_DEX_H_BOX_HELICASES_ACTIVATE_TYPE_I_IFN_AND_INFLAMMATORY_CYTOKINES_PRODUCTION	28.57	9.38E-04	down
REACTOME_RHO_GTPASES_ACTIVATE_NADPH_OXIDASES	20.83	5.11E-07	down
REACTOME_THE_NLRP3_INFLAMMASOME	20.00	1.31E-04	down
REACTOME_ACTIVATION_OF_THE_AP_1_FAMILY_OF_TRANSCRIPTION_FACTORS	20.00	1.98E-03	down
REACTOME_ALTERNATIVE_COMPLEMENT_ACTIVATION	20.00	3.35E-02	down
REACTOME_CD22_MEDIATED_BCR_REGULATION	20.00	3.35E-02	down
REACTOME_FICOLINS_BIND_TO_REPETITIVE_CARBOHYDRATE_STRUCTURES_ON_THE_TARGET_CELL_SURFACE	20.00	3.35E-02	down
REACTOME_LEUKOTRIENE_RECEPTORS	20.00	3.35E-02	down
REACTOME_CREB1_PHOSPHORYLATION_THROUGH_THE_ACTIVATION_OF_ADENYLATE_CYCLASE	16.67	9.85E-03	up
REACTOME_ROS_AND_RNS_PRODUCTION_IN_PHAGOCYTES	16.67	1.44E-07	down
REACTOME_FCGR_ACTIVATION	16.67	2.88E-03	down
REACTOME_PROTEIN_REPAIR	16.67	4.00E-02	down
REACTOME_PROTON_COUPLED_MONOCARBOXYLATE_TRANSPORT	16.67	4.00E-02	down
REACTOME_RUNX3_REGULATES_IMMUNE_RESPONSE_AND_CELL_MIGRATION	16.67	4.00E-02	down
REACTOME_VLDL_CLEARANCE	16.67	4.00E-02	down
REACTOME_DISSOLUTION_OF_FIBRIN_CLOT	15.38	3.39E-03	down
REACTOME_SCAVENGING_OF_HEME_FROM_PLASMA	15.38	3.39E-03	down
REACTOME_INFLAMMASOMES	15.00	3.20E-04	down
REACTOME_ACTIVATION_OF_C3_AND_C5	14.29	4.65E-02	down
REACTOME_ACTIVATION_OF_RAC1_DOWNSTREAM_OF_NMDARS	14.29	4.65E-02	down
REACTOME_ATTACHMENT_OF_GPI_ANCHOR_TO_UPAR	14.29	4.65E-02	down
REACTOME_IKBA_VARIANT_LEADS_TO_EDA_ID	14.29	4.65E-02	down
REACTOME_PHOSPHATE_BOND_HYDROLYSIS_BY_NUDT_PROTEINS	14.29	4.65E-02	down
REACTOME_RUNX3_REGULATES_CDKN1A_TRANSCRIPTION	14.29	4.65E-02	down
REACTOME_RAF_INDEPENDENT_MAPK1_3_ACTIVATION	13.04	4.90E-04	down
REACTOME_PKA_ACTIVATION_IN_GLUCAGON_SIGNALLING	11.76	1.95E-02	up
REACTOME_PROTEIN_METHYLATION	11.76	1.95E-02	up
REACTOME_BRANCHED_CHAIN_AMINO_ACID_CATABOLISM	10.53	2.41E-02	up
REACTOME_CONVERSION_FROM_APC_C:CDC20_TO_APC_C:CDH1_IN_LATE_ANAPHASE	10.53	2.41E-02	up
REACTOME_PKA_MEDIATED_PHOSPHORYLATION_OF_CREB	10.53	2.41E-02	up
REACTOME_OTHER_SEMAPHORIN_INTERACTIONS	10.53	7.24E-03	down
REACTOME_REGULATION_OF_TLR_BY_ENDOGENOUS_LIGAND	10.53	7.24E-03	down
REACTOME_SIGNALING_BY_HIPPO	10.00	2.65E-02	up
REACTOME_TP53_REGULATES_TRANSCRIPTION_OF_ADDITIONAL_CELL_CYCLE_GENES_WHOSE_EXACT_ROLE_IN_THE_P53_PATHWAY_REMAIN_UNCERTAIN	9.52	8.82E-03	down
REACTOME_FCERI_MEDIATED_MAPK_ACTIVATION	9.38	1.31E-03	down
REACTOME_INITIAL_TRIGGERING_OF_COMPLEMENT	9.09	9.66E-03	down
REACTOME_BBSOME_MEDIATED_CARGO_TARGETING_TO_CILIUM	8.70	3.44E-02	up
REACTOME_DARPP_32_EVENTS	8.33	3.73E-02	up
REACTOME_DISEASES_OF_IMMUNE_SYSTEM	8.33	1.14E-02	down
REACTOME_SEMA4D_IN_SEMAPHORIN_SIGNALING	8.33	1.14E-02	down
REACTOME_RETROGRADE_TRANSPORT_AT_THE_TRANS_GOLGI_NETWORK	8.16	3.50E-03	up
REACTOME_DETOXIFICATION_OF_REACTIVE_OXYGEN_SPECIES	8.11	2.00E-03	down
REACTOME_SURFACTANT_METABOLISM	6.90	1.65E-02	down

REACTOME_MAPK_TARGETS_NUCLEAR_EVENTS_MEDIATED_BY_MAP_KINASES	6.45	1.87E-02	down
REACTOME_PYRUVATE_METABOLISM	6.45	1.87E-02	down
REACTOME_NEUTROPHIL_DEGRANULATION	6.28	9.76E-21	down
REACTOME_SEMAPHORIN_INTERACTIONS	6.25	9.40E-04	down
REACTOME_INTERFERON_ALPHA_BETA_SIGNALING	5.80	1.25E-03	down
REACTOME_GPVI_MEDIATED_ACTIVATION_CASCADE	5.71	2.35E-02	down
REACTOME_INTRAFLAGELLAR_TRANSPORT	5.56	3.16E-02	up
REACTOME_NUCLEOTIDE_BINDING_DOMAIN_LEUCINE_RICH_REPEAT_CONTAINI NG_RECEPTOR_NLR_SIGNALING_PATHWAYS	5.56	5.89E-03	down
REACTOME_NUCLEOBASE_CATABOLISM	5.56	2.48E-02	down
REACTOME_O_GLYCOSYLATION_OF_TSR_DOMAIN_CONTAINING_PROTEINS	5.26	2.74E-02	down
REACTOME_ANCHORING_OF_THE_BASAL_BODY_TO_THE_PLASMA_MEMBRANE	5.15	8.10E-03	up
REACTOME_NEGATIVE_REGULATION_OF_MAPK_PATHWAY	5.00	3.01E-02	down
REACTOME_CILIUM_ASSEMBLY	4.98	2.70E-04	up
REACTOME_REGULATION_OF_INSULIN_LIKE_GROWTH_FACTOR_IGF_TRANSPOR T_AND_UPTAKE_BY_INSULIN_LIKE_GROWTH_FACTOR_BINDING_PROTEINS_IGFB PS	4.84	2.02E-04	down
REACTOME_BINDING_AND_UPTAKE_OF_LIGANDS_BY_SCAVENGER_RECEPTORS	4.76	3.30E-02	down
REACTOME_TOLL_LIKE_RECEPTOR_CASCADES	4.58	8.43E-05	down
REACTOME_INTERLEUKIN_10_SIGNALING	4.35	3.89E-02	down
REACTOME_RHO_GTPASE_CYCLE	4.32	8.95E-03	up
REACTOME_INTERLEUKIN_17_SIGNALING	4.23	1.25E-02	down
REACTOME_TOLL_LIKE_RECEPTOR_9_TLR9_CASCADE	4.21	4.01E-03	down
REACTOME_TRIF_TICAM1_MEDIATED_TLR4_SIGNALING	4.12	4.31E-03	down
REACTOME_TP53_REGULATES_TRANSCRIPTION_OF_CELL_CYCLE_GENES	4.08	4.37E-02	down
REACTOME_ORGANELLE_BIOGENESIS_AND_MAINTENANCE	4.07	4.33E-04	up
REACTOME_IMMUNOREGULATORY_INTERACTIONS_BETWEEN_A_LYMPHOID_AN D_A_NON_LYMPHOID_CELL	3.94	1.75E-03	down
REACTOME_DDX58_IFIH1_MEDIATED_INDUCTION_OF_INTERFERON_ALPHA_BET A	3.85	1.61E-02	down
REACTOME_INNATE_IMMUNE_SYSTEM	3.83	7.94E-20	down
REACTOME_INTERLEUKIN_4_AND_INTERLEUKIN_13_SIGNALING	3.70	6.30E-03	down
REACTOME_CELL_SURFACE_INTERACTIONS_AT_THE_VASCULAR_WALL	3.65	2.44E-03	down
REACTOME_MYD88_CASCADE_INITIATED_ON_PLASMA_MEMBRANE	3.57	1.95E-02	down
REACTOME_TRANSCRIPTIONAL_REGULATION_OF_WHITE_ADIPOCYTE_DIFFERE NTIATION	3.57	1.95E-02	down
REACTOME_TRANSCRIPTIONAL_REGULATION_OF_GRANULOPOIESIS	3.41	2.21E-02	down
REACTOME_TOLL_LIKE_RECEPTOR_4_TLR4_CASCADE	3.15	1.10E-02	down
REACTOME_ANTIMICROBIAL_PEPTIDES	3.13	2.76E-02	down
REACTOME_TOLL_LIKE_RECEPTOR_TLR1:TLR2_CASCADE	3.09	2.84E-02	down
REACTOME_ANTIGEN_PROCESSING_CROSS_PRESENTATION	3.03	2.99E-02	down
REACTOME_TNFR2_NON_CANONICAL_NF_KB_PATHWAY	3.00	3.07E-02	down
REACTOME_SIGNALING_BY_VEGF	2.86	3.47E-02	down
REACTOME_SENESCENCE_ASSOCIATED_SECRETORY_PHENOTYPE_SASP	2.78	3.72E-02	down
REACTOME_GENERIC_TRANSCRIPTION_PATHWAY	2.14	7.07E-03	up
REACTOME_CYTOKINE_SIGNALING_IN_IMMUNE_SYSTEM	2.12	1.80E-05	down
REACTOME_SIGNALING_BY_INTERLEUKINS	2.07	3.51E-04	down
REACTOME_INTERFERON_SIGNALING	2.01	4.68E-02	down
REACTOME_HEMOSTASIS	1.94	1.02E-03	down
REACTOME_RHO_GTPASE_EFFECTORS	1.87	2.24E-02	down
REACTOME_GENE_EXPRESSION_TRANSCRIPTION	1.83	3.81E-02	up
REACTOME_CELLULAR_RESPONSES_TO_STRESS	1.60	3.00E-02	down
REACTOME_ADAPTIVE_IMMUNE_SYSTEM	1.47	1.30E-02	down

Table S4. All DEG in comparison of (pediatric CML vs pediatric healthy) vs (adult CML vs adult healthy), sorted according to logFC level. *p* values were obtained using hypergeometric test. In red are genes that we validated by RT-qPCR.

**Upregulated in Pediatric CML Compared to Adult CML**				**Downregulated in Pediatric CML Compared to Adult CML**			
**Gene**	**logFC**	***p*.Value**	**adj.*p*.Val**	**Gene**	**logFC**	***p*.Value**	**adj.*p*.Val**
PLOD2	4.19	1.30E-03	6.44E-02	HM13	-1.01	3.70E-03	8.19E-02
MYCT1	4.18	0.00E+00	4.06E-02	SLC43A3	-1.01	9.50E-03	1.10E-01
CD40LG	3.88	9.00E-04	5.99E-02	ACAA1	-1.02	1.30E-03	6.43E-02
SH3BGRL2	3.63	1.40E-03	6.53E-02	TANGO2	-1.02	1.60E-03	6.69E-02
FAM171A2	3.55	5.30E-03	9.11E-02	SYK	-1.02	1.80E-03	6.73E-02
WDR44	3.52	2.00E-04	4.42E-02	LAMTOR2	-1.02	8.80E-03	1.08E-01
MYEF2	3.51	1.00E-04	4.16E-02	RHOG	-1.03	7.20E-03	1.01E-01
PKIA	3.51	2.00E-04	4.61E-02	NUBP1	-1.04	1.10E-03	6.35E-02
FAM199X	3.47	3.00E-04	5.12E-02	STX10	-1.04	4.40E-03	8.74E-02
FREM1	3.47	7.90E-03	1.05E-01	DNAJB1	-1.04	7.40E-03	1.01E-01
CLDN10	3.45	5.00E-04	5.64E-02	CPTP	-1.04	8.40E-03	1.06E-01
CEP70	3.42	0.00E+00	4.06E-02	SNX20	-1.04	1.02E-02	1.12E-01
BEND4	3.42	1.40E-03	6.48E-02	GNB2	-1.05	7.00E-03	1.00E-01
LYSMD3	3.38	8.00E-04	5.85E-02	BORCS6	-1.06	7.30E-03	1.01E-01
BMI1	3.37	8.00E-04	5.85E-02	HLA.DPA1	-1.06	1.00E-02	1.12E-01
VANGL2	3.37	5.10E-03	9.01E-02	HPCAL1	-1.07	2.70E-03	7.38E-02
SLC24A3	3.36	1.90E-03	6.76E-02	IL27RA	-1.08	7.30E-03	1.01E-01
MYCN	3.33	2.90E-03	7.64E-02	VPS18	-1.09	2.30E-03	7.13E-02
WFDC1	3.32	2.70E-03	7.38E-02	TOLLIP	-1.09	3.00E-03	7.79E-02
PEX7	3.29	1.10E-03	6.25E-02	PLOD1	-1.09	4.80E-03	8.88E-02
SGPP1	3.29	2.10E-03	6.85E-02	PLD3	-1.09	5.50E-03	9.19E-02
SVOPL	3.28	8.00E-04	5.85E-02	TRIOBP	-1.1	1.60E-03	6.73E-02
TTK	3.26	2.90E-03	7.64E-02	KIAA0922	-1.1	6.50E-03	9.78E-02
ZNF675	3.24	5.00E-04	5.61E-02	PTPN18	-1.1	6.80E-03	9.95E-02
FKBP14	3.18	1.60E-03	6.73E-02	NUP214	-1.1	8.80E-03	1.08E-01
OXR1	3.14	7.00E-04	5.85E-02	CCM2	-1.11	7.00E-04	5.85E-02
FNBP1L	3.09	1.30E-03	6.43E-02	MFSD12	-1.11	1.90E-03	6.77E-02
SCOC	3.07	4.00E-04	5.14E-02	PARVG	-1.11	5.00E-03	8.94E-02
SERAC1	3	1.00E-04	4.06E-02	RPS6KA1	-1.12	1.00E-03	6.16E-02
FAM160B1	3	2.00E-03	6.85E-02	MARK2	-1.12	1.50E-03	6.62E-02
NUDCD1	3	8.60E-03	1.07E-01	HLA.DPB1	-1.12	3.70E-03	8.18E-02
ZSCAN12	2.99	1.10E-03	6.33E-02	GALNS	-1.12	1.12E-02	1.17E-01
TMEM163	2.98	7.30E-03	1.01E-01	PQLC1	-1.13	1.60E-03	6.69E-02
NEMP2	2.96	1.10E-03	6.33E-02	LAIR1	-1.13	4.50E-03	8.76E-02
ZNF776	2.94	6.00E-04	5.80E-02	APLP2	-1.13	4.60E-03	8.76E-02
GALNT12	2.93	1.80E-03	6.76E-02	RNF135	-1.13	6.60E-03	9.79E-02
ZNF493	2.92	2.00E-04	4.42E-02	SPINT1	-1.14	4.50E-03	8.76E-02
DIRAS1	2.92	2.10E-03	6.85E-02	SLC27A1	-1.14	8.00E-03	1.05E-01
ZNF136	2.91	1.30E-03	6.48E-02	TWF2	-1.14	9.70E-03	1.11E-01
DNAJC18	2.89	4.00E-04	5.14E-02	SUN2	-1.15	6.50E-03	9.78E-02
TADA1	2.89	9.00E-04	5.88E-02	TRADD	-1.16	2.10E-03	6.85E-02
HOXB2	2.88	1.90E-03	6.76E-02	ABHD8	-1.16	5.00E-03	8.94E-02
C12orf66	2.87	1.70E-03	6.73E-02	LSM10	-1.16	6.50E-03	9.78E-02
ZNF555	2.87	4.80E-03	8.87E-02	C20orf27	-1.16	8.80E-03	1.08E-01
IGF2BP2	2.86	6.70E-03	9.91E-02	EHD1	-1.16	9.20E-03	1.10E-01
MTHFD2L	2.84	8.00E-04	5.85E-02	ACTB	-1.17	5.30E-03	9.11E-02
FANCF	2.83	6.30E-03	9.76E-02	ITPK1	-1.17	8.30E-03	1.06E-01
RRM2B	2.81	3.30E-03	7.88E-02	SLC3A2	-1.18	1.00E-04	4.42E-02
EXTL2	2.81	4.90E-03	8.88E-02	LRPAP1	-1.18	7.00E-04	5.80E-02
THRB	2.79	9.00E-04	5.88E-02	SHKBP1	-1.18	1.90E-03	6.78E-02

PLEKHA4	2.78	4.10E-03	8.59E-02	QSOX1	-1.18	4.40E-03	8.74E-02
SWT1	2.78	5.70E-03	9.29E-02	APOL3	-1.18	5.20E-03	9.10E-02
USP46	2.76	6.00E-04	5.80E-02	NADK	-1.19	6.00E-04	5.80E-02
SASS6	2.76	2.40E-03	7.14E-02	LENG1	-1.19	2.20E-03	6.92E-02
MBTPS2	2.76	3.90E-03	8.43E-02	CHCHD10	-1.19	7.40E-03	1.01E-01
TMEM231	2.75	4.50E-03	8.74E-02	PIK3CD	-1.19	8.10E-03	1.05E-01
ZNF569	2.72	9.00E-04	5.85E-02	PPP1R18	-1.2	1.50E-03	6.65E-02
WDR35	2.69	8.00E-04	5.85E-02	LTBR	-1.2	3.30E-03	7.86E-02
C5	2.66	1.20E-03	6.43E-02	MAN2B1	-1.2	5.80E-03	9.36E-02
KLHL28	2.62	1.00E-03	6.18E-02	CBX6	-1.2	8.30E-03	1.06E-01
ZNF549	2.62	8.40E-03	1.07E-01	CCDC137	-1.2	9.50E-03	1.10E-01
PCYT1B	2.61	6.10E-03	9.62E-02	RNF122	-1.2	1.03E-02	1.12E-01
PACRGL	2.6	3.90E-03	8.42E-02	PAK1	-1.21	2.10E-03	6.85E-02
NAA30	2.58	2.10E-03	6.85E-02	SRA1	-1.21	5.30E-03	9.11E-02
DCUN1D1	2.57	1.50E-03	6.62E-02	DBNL	-1.24	8.00E-04	5.85E-02
RNF217	2.56	5.10E-03	9.01E-02	DESI1	-1.25	1.60E-03	6.69E-02
KPNA5	2.55	1.80E-03	6.76E-02	MAP3K11	-1.25	2.60E-03	7.32E-02
ZNF518B	2.55	2.60E-03	7.37E-02	TRIM8	-1.25	6.40E-03	9.78E-02
TRO	2.54	1.11E-02	1.17E-01	ZDHHC12	-1.26	3.00E-04	4.61E-02
PBX1	2.53	2.80E-03	7.50E-02	GMPPB	-1.26	1.60E-03	6.73E-02
MPP6	2.53	3.30E-03	7.86E-02	OAZ1	-1.26	5.40E-03	9.18E-02
CEP83	2.53	3.40E-03	7.92E-02	MYO1G	-1.26	5.90E-03	9.44E-02
TRIB2	2.53	6.80E-03	9.91E-02	FRAT1	-1.26	9.90E-03	1.11E-01
ZNF254	2.52	7.00E-04	5.85E-02	GPSM3	-1.28	3.20E-03	7.79E-02
PLCB4	2.52	2.40E-03	7.15E-02	FRAT2	-1.28	4.90E-03	8.88E-02
PIGZ	2.51	1.07E-02	1.15E-01	SNX8	-1.29	0.00E+00	4.06E-02
CCDC171	2.5	5.80E-03	9.36E-02	PTK2B	-1.29	3.00E-04	4.87E-02
OMA1	2.49	2.20E-03	7.02E-02	LIMK1	-1.29	4.20E-03	8.59E-02
SOCS6	2.47	2.20E-03	7.01E-02	NEK6	-1.29	6.40E-03	9.78E-02
XPR1	2.47	2.50E-03	7.27E-02	XKR8	-1.29	9.70E-03	1.10E-01
H1F0	2.46	3.40E-03	7.92E-02	RIN3	-1.3	2.40E-03	7.14E-02
TOM1L1	2.44	2.70E-03	7.38E-02	RENBP	-1.3	1.05E-02	1.14E-01
SPAG16	2.44	9.10E-03	1.09E-01	DOK3	-1.31	1.30E-03	6.43E-02
METTL10	2.43	3.60E-03	8.18E-02	POR	-1.31	3.20E-03	7.86E-02
CGRRF1	2.43	1.08E-02	1.16E-01	MAP2K3	-1.32	1.00E-04	4.06E-02
MEIS1	2.42	7.30E-03	1.01E-01	CDK2AP2	-1.32	4.00E-04	5.16E-02
GSTCD	2.41	6.30E-03	9.78E-02	RNPEPL1	-1.32	1.00E-03	6.09E-02
USP51	2.41	1.06E-02	1.15E-01	HCLS1	-1.32	2.40E-03	7.14E-02
ZNF521	2.39	1.60E-03	6.69E-02	HLA.DQA1	-1.32	7.10E-03	1.01E-01
ATF7IP2	2.39	2.60E-03	7.32E-02	FAM110A	-1.32	9.70E-03	1.10E-01
EPHX2	2.39	7.80E-03	1.04E-01	UCP2	-1.33	4.00E-04	5.16E-02
ZNF614	2.39	7.90E-03	1.04E-01	S1PR4	-1.33	7.00E-04	5.85E-02
ACTR6	2.38	2.10E-03	6.85E-02	GNAI2	-1.34	2.20E-03	7.02E-02
FAM83D	2.38	6.70E-03	9.88E-02	WAS	-1.35	6.00E-04	5.80E-02
MIER3	2.37	6.70E-03	9.88E-02	SDF2L1	-1.35	8.00E-04	5.85E-02
SMO	2.37	8.00E-03	1.05E-01	FAM65B	-1.35	3.90E-03	8.43E-02
CHRM3	2.37	1.06E-02	1.14E-01	SLC38A10	-1.37	1.00E-04	4.06E-02
LRRC37B	2.36	5.30E-03	9.10E-02	SLC9A1	-1.37	2.00E-03	6.85E-02
AASDH	2.36	6.30E-03	9.78E-02	TSPAN14	-1.38	4.00E-04	5.14E-02
ZNF736	2.34	5.90E-03	9.41E-02	GPX1	-1.38	4.20E-03	8.70E-02
ARHGAP18	2.32	5.50E-03	9.23E-02	CYTH1	-1.39	3.60E-03	8.18E-02
SEC23A	2.3	7.40E-03	1.02E-01	SP110	-1.39	9.80E-03	1.11E-01
CLGN	2.3	9.50E-03	1.10E-01	HLA.C	-1.39	1.12E-02	1.17E-01
COG6	2.3	1.15E-02	1.19E-01	CNPY3	-1.4	2.00E-04	4.42E-02

UACA	2.28	6.00E-03	9.50E-02	FGD3	-1.4	2.50E-03	7.27E-02
CLC	2.28	1.12E-02	1.17E-01	PRKACA	-1.4	2.70E-03	7.38E-02
SERPINE2	2.26	4.00E-04	5.16E-02	HLA.DRA	-1.4	3.30E-03	7.86E-02
TPD52	2.25	1.00E-03	6.18E-02	TBC1D2	-1.4	5.00E-03	8.94E-02
TRIM59	2.25	2.10E-03	6.85E-02	SEPT9	-1.41	3.20E-03	7.79E-02
ZNF880	2.23	4.40E-03	8.71E-02	ARHGAP45	-1.41	5.80E-03	9.36E-02
MED21	2.23	8.80E-03	1.08E-01	TTC7A	-1.42	1.40E-03	6.53E-02
PINLYP	2.23	9.10E-03	1.09E-01	CST7	-1.42	2.70E-03	7.38E-02
ZSCAN9	2.22	5.90E-03	9.41E-02	NEU1	-1.43	1.60E-03	6.69E-02
ZNF329	2.22	7.10E-03	1.01E-01	REEP4	-1.43	1.70E-03	6.73E-02
TMX3	2.22	1.05E-02	1.14E-01	ABR	-1.43	4.30E-03	8.70E-02
POLE2	2.21	2.30E-03	7.02E-02	PXN	-1.43	4.90E-03	8.89E-02
TMEM237	2.21	3.50E-03	8.08E-02	CYB561D2	-1.43	7.40E-03	1.01E-01
KIF27	2.21	1.02E-02	1.12E-01	LPCAT1	-1.43	1.01E-02	1.12E-01
BBS9	2.2	9.70E-03	1.10E-01	TMSB10	-1.43	1.03E-02	1.13E-01
ZBTB34	2.2	1.09E-02	1.16E-01	SCO2	-1.44	8.70E-03	1.08E-01
IFT81	2.19	6.30E-03	9.76E-02	ATP6V0B	-1.44	1.11E-02	1.17E-01
ZNF256	2.18	9.90E-03	1.11E-01	RILPL2	-1.45	5.50E-03	9.19E-02
RCN2	2.17	1.15E-02	1.19E-01	DOCK10	-1.45	9.50E-03	1.10E-01
MFN1	2.16	7.40E-03	1.01E-01	AKAP13	-1.45	9.70E-03	1.10E-01
PIR	2.16	7.70E-03	1.04E-01	PDXK	-1.45	1.06E-02	1.15E-01
LEPR	2.14	1.40E-03	6.53E-02	MCOLN1	-1.46	2.60E-03	7.32E-02
GAN	2.14	4.90E-03	8.88E-02	PKM	-1.46	4.00E-03	8.50E-02
C9orf85	2.14	6.70E-03	9.88E-02	ROGDI	-1.46	5.90E-03	9.41E-02
METTL21B	2.13	3.10E-03	7.79E-02	RPS6KA4	-1.47	3.00E-04	4.99E-02
ABCC4	2.12	2.00E-04	4.61E-02	LAPTM5	-1.47	2.90E-03	7.60E-02
STAT4	2.12	3.20E-03	7.79E-02	GMIP	-1.47	3.30E-03	7.89E-02
ZNF711	2.11	3.50E-03	7.96E-02	RCSD1	-1.47	8.10E-03	1.05E-01
NEDD4	2.11	4.90E-03	8.88E-02	HS1BP3	-1.48	1.70E-03	6.73E-02
FHIT	2.11	1.14E-02	1.18E-01	HOOK2	-1.48	4.60E-03	8.76E-02
NUP54	2.1	3.40E-03	7.92E-02	MYD88	-1.48	8.80E-03	1.08E-01
IMPA1	2.1	9.20E-03	1.09E-01	PREX1	-1.49	3.00E-04	4.61E-02
MTRF1L	2.1	1.02E-02	1.12E-01	ANXA6	-1.49	3.70E-03	8.18E-02
ITGB3BP	2.09	6.10E-03	9.64E-02	ABCD1	-1.49	7.20E-03	1.01E-01
SPATA7	2.08	2.30E-03	7.08E-02	RNASET2	-1.49	9.50E-03	1.10E-01
VCPKMT	2.07	8.90E-03	1.08E-01	ESRRA	-1.5	2.00E-04	4.42E-02
CCSAP	2.07	1.03E-02	1.12E-01	C17orf62	-1.5	1.90E-03	6.77E-02
C2orf69	2.07	1.08E-02	1.16E-01	OGFR	-1.53	4.00E-04	5.14E-02
TMOD1	2.06	4.00E-04	5.14E-02	SH3BP1	-1.53	3.90E-03	8.43E-02
DYNLT3	2.06	1.13E-02	1.17E-01	E2F2	-1.53	4.50E-03	8.74E-02
CEP97	2.05	4.30E-03	8.70E-02	GOLGA3	-1.53	4.80E-03	8.87E-02
ZNF124	2.05	1.04E-02	1.13E-01	GM2A	-1.53	9.20E-03	1.10E-01
LRRC40	2.02	6.80E-03	9.98E-02	TEP1	-1.53	9.60E-03	1.10E-01
KCTD9	2.02	9.10E-03	1.09E-01	TMEM8A	-1.54	0.00E+00	4.06E-02
RBM45	2.01	8.70E-03	1.08E-01	TAP1	-1.55	2.00E-04	4.61E-02
BEX2	2.01	1.00E-02	1.12E-01	ARAP1	-1.56	2.00E-04	4.42E-02
ZNF141	2	1.50E-03	6.65E-02	RNH1	-1.57	7.00E-04	5.85E-02
MECOM	1.99	1.50E-03	6.62E-02	IMPDH1	-1.58	0.00E+00	4.06E-02
SLAIN1	1.99	1.13E-02	1.17E-01	RNF213	-1.58	3.60E-03	8.18E-02
BCKDHB	1.98	4.40E-03	8.74E-02	IL4R	-1.58	8.70E-03	1.08E-01
PRPF18	1.98	1.10E-02	1.17E-01	HLA.DRB1	-1.59	1.80E-03	6.76E-02
FGGY	1.96	9.30E-03	1.10E-01	SMAP2	-1.59	3.10E-03	7.79E-02
TFPI	1.95	6.00E-04	5.80E-02	NBEAL2	-1.59	8.70E-03	1.08E-01
TRUB1	1.95	8.50E-03	1.07E-01	TOM1	-1.59	9.30E-03	1.10E-01

CFAP97	1.94	1.00E-03	6.16E-02	UNC119	-1.6	1.50E-03	6.62E-02
TTC26	1.94	5.90E-03	9.45E-02	DTX2	-1.61	1.20E-03	6.43E-02
HACD1	1.93	2.90E-03	7.64E-02	CCDC142	-1.61	5.50E-03	9.21E-02
MBIP	1.93	2.90E-03	7.64E-02	MID1IP1	-1.62	6.00E-04	5.80E-02
MED12L	1.92	9.90E-03	1.11E-01	ARRB2	-1.62	4.70E-03	8.83E-02
GATA2	1.92	1.10E-02	1.16E-01	SH3BP2	-1.62	6.40E-03	9.78E-02
PEX1	1.91	8.80E-03	1.08E-01	WARS	-1.63	1.10E-03	6.35E-02
BDH2	1.89	3.50E-03	8.09E-02	ATP6V0D1	-1.63	3.90E-03	8.42E-02
**DLC1**	1.89	5.10E-03	9.01E-02	TLE4	-1.63	8.30E-03	1.06E-01
RHOBTB1	1.89	5.80E-03	9.36E-02	APOL1	-1.63	1.08E-02	1.15E-01
GPHN	1.89	7.30E-03	1.01E-01	MGAT1	-1.64	1.00E-04	4.42E-02
CASD1	1.87	3.00E-04	5.12E-02	HLA.B	-1.64	1.20E-03	6.43E-02
CCNT1	1.87	8.50E-03	1.07E-01	ABTB1	-1.64	2.00E-03	6.85E-02
RIMKLB	1.87	9.10E-03	1.09E-01	RAB11FIP1	-1.64	4.70E-03	8.78E-02
CNRIP1	1.86	1.90E-03	6.78E-02	ARHGAP30	-1.65	2.10E-03	6.86E-02
TMEM64	1.86	2.20E-03	6.98E-02	DEF8	-1.65	2.20E-03	7.01E-02
YEATS4	1.86	1.05E-02	1.14E-01	C15orf39	-1.67	1.00E-04	4.06E-02
EOGT	1.85	9.70E-03	1.11E-01	EMILIN2	-1.68	4.70E-03	8.78E-02
MAP9	1.84	7.10E-03	1.01E-01	JMJD6	-1.69	2.30E-03	7.13E-02
NR1D2	1.84	8.00E-03	1.05E-01	SLC35F6	-1.69	4.10E-03	8.59E-02
C12orf29	1.83	5.70E-03	9.29E-02	HLA.A	-1.71	1.20E-03	6.43E-02
BMT2	1.83	9.60E-03	1.10E-01	SPI1	-1.72	3.00E-03	7.76E-02
ZNF551	1.83	1.12E-02	1.17E-01	PECAM1	-1.72	4.60E-03	8.76E-02
PAWR	1.82	2.70E-03	7.38E-02	BASP1	-1.72	6.80E-03	9.95E-02
MORC3	1.82	7.80E-03	1.04E-01	TMEM175	-1.74	1.40E-03	6.53E-02
PARP11	1.81	6.00E-03	9.50E-02	IL10RB	-1.74	4.80E-03	8.88E-02
PDZD8	1.8	2.40E-03	7.17E-02	SAT2	-1.74	5.00E-03	9.00E-02
C2orf88	1.8	6.10E-03	9.64E-02	NLRC5	-1.74	7.10E-03	1.01E-01
PGBD1	1.8	6.70E-03	9.90E-02	ALDH3B1	-1.74	8.40E-03	1.06E-01
CENPK	1.8	9.90E-03	1.11E-01	PYCARD	-1.75	1.40E-03	6.48E-02
ZNF765	1.79	7.30E-03	1.01E-01	FAM50A	-1.75	1.90E-03	6.76E-02
MLLT3	1.78	0.00E+00	4.06E-02	CD74	-1.76	1.00E-04	4.06E-02
ST8SIA6	1.78	1.30E-03	6.43E-02	FBXO6	-1.76	1.15E-02	1.19E-01
SENP7	1.78	8.50E-03	1.07E-01	IL16	-1.77	2.50E-03	7.19E-02
STK3	1.78	1.02E-02	1.12E-01	ARPC1B	-1.78	1.10E-03	6.39E-02
ACADSB	1.77	6.90E-03	1.00E-01	PARD6A	-1.78	2.40E-03	7.14E-02
PRKCI	1.72	1.70E-03	6.73E-02	EHBP1L1	-1.78	5.10E-03	9.01E-02
CYYR1	1.72	6.90E-03	1.00E-01	AP5B1	-1.79	1.50E-03	6.62E-02
RRAGB	1.71	7.70E-03	1.03E-01	PQLC2	-1.8	1.70E-03	6.73E-02
ALDH1A1	1.71	8.30E-03	1.06E-01	SH2D3C	-1.81	1.80E-03	6.76E-02
RABL2A	1.71	9.50E-03	1.10E-01	SLC12A9	-1.82	1.00E-04	4.42E-02
DUS4L	1.7	1.06E-02	1.15E-01	NAGA	-1.82	3.70E-03	8.26E-02
PDK1	1.69	6.90E-03	1.00E-01	NUDT16	-1.82	4.00E-03	8.47E-02
TTC7B	1.68	8.00E-04	5.85E-02	SH3BGRL3	-1.82	6.00E-03	9.55E-02
GSKIP	1.68	7.60E-03	1.03E-01	EFHD2	-1.84	6.00E-04	5.77E-02
C1orf21	1.66	1.00E-03	6.16E-02	TMSB4X	-1.84	3.60E-03	8.18E-02
BEX3	1.66	1.40E-03	6.48E-02	ALDH2	-1.84	4.20E-03	8.70E-02
ACSM3	1.66	1.40E-03	6.53E-02	TIMP2	-1.84	5.00E-03	8.94E-02
STXBP5	1.64	2.00E-04	4.61E-02	HERPUD1	-1.85	2.00E-03	6.85E-02
MTURN	1.62	0.00E+00	4.06E-02	CCDC88C	-1.85	7.20E-03	1.01E-01
ZNF43	1.61	3.00E-04	5.12E-02	HDAC4	-1.86	5.00E-04	5.64E-02
RP11.382A20.3	1.6	3.80E-03	8.28E-02	FTL	-1.86	6.50E-03	9.78E-02
PRKACB	1.6	4.50E-03	8.74E-02	CCND3	-1.88	0.00E+00	4.06E-02
CDADC1	1.6	4.60E-03	8.76E-02	SULT1A1	-1.88	8.90E-03	1.08E-01

ORC3	1.6	1.11E-02	1.17E-01	ADGRE5	-1.89	3.10E-03	7.79E-02
ZNF512	1.59	3.00E-04	4.61E-02	CD53	-1.91	9.00E-04	5.85E-02
CSNK1G3	1.59	1.01E-02	1.12E-01	S100A4	-1.93	9.40E-03	1.10E-01
CUL5	1.58	2.00E-04	4.42E-02	SLC27A3	-1.94	9.00E-04	5.88E-02
MCTP2	1.58	8.00E-04	5.85E-02	CD52	-1.94	6.20E-03	9.72E-02
CD2AP	1.56	1.40E-03	6.53E-02	CITED4	-1.96	6.00E-04	5.80E-02
AGL	1.56	9.00E-03	1.09E-01	AKNA	-1.96	2.70E-03	7.38E-02
CDK17	1.55	8.00E-04	5.85E-02	SH2B2	-1.96	1.16E-02	1.19E-01
ARHGAP5	1.55	5.50E-03	9.23E-02	MOB3A	-1.97	2.00E-04	4.42E-02
DEPTOR	1.55	6.60E-03	9.78E-02	CTSA	-1.97	3.00E-04	5.12E-02
UFSP2	1.55	9.90E-03	1.11E-01	CYBA	-1.97	9.00E-04	5.85E-02
HOMER2	1.54	0.00E+00	4.06E-02	RGS19	-1.98	7.00E-04	5.85E-02
CKAP2	1.54	4.60E-03	8.78E-02	SEMA4B	-1.98	1.50E-03	6.62E-02
TWSG1	1.54	4.70E-03	8.83E-02	HLX	-1.98	2.10E-03	6.85E-02
IMMP2L	1.54	8.00E-03	1.05E-01	ZDHHC1	-1.98	2.90E-03	7.60E-02
EPSTI1	1.53	1.00E-04	4.06E-02	TSC22D3	-1.98	3.70E-03	8.24E-02
GTDC1	1.53	3.90E-03	8.43E-02	CACFD1	-1.99	8.00E-04	5.85E-02
LRBA	1.52	4.10E-03	8.55E-02	TUBA4A	-1.99	1.30E-03	6.44E-02
PHF6	1.52	5.30E-03	9.10E-02	LTB4R	-1.99	5.10E-03	9.01E-02
N4BP2	1.51	8.00E-04	5.85E-02	**ARHGAP27**	-2.02	6.00E-04	5.80E-02
DNAJB14	1.5	1.90E-03	6.76E-02	BRI3	-2.02	3.90E-03	8.43E-02
ERMP1	1.5	7.20E-03	1.01E-01	SPON2	-2.03	3.60E-03	8.18E-02
ASB9	1.49	2.90E-03	7.64E-02	CORO1A	-2.06	1.00E-04	4.16E-02
STAP1	1.48	3.20E-03	7.79E-02	SCPEP1	-2.06	1.02E-02	1.12E-01
NCK1	1.46	4.80E-03	8.88E-02	GRINA	-2.08	2.00E-04	4.61E-02
HOXA9	1.45	1.70E-03	6.73E-02	GAA	-2.08	1.10E-03	6.33E-02
STAM	1.45	5.30E-03	9.10E-02	MVP	-2.08	2.50E-03	7.24E-02
KIF2A	1.45	5.80E-03	9.36E-02	ZFHX3	-2.08	4.50E-03	8.74E-02
CDK6	1.44	9.40E-03	1.10E-01	BTG1	-2.09	1.70E-03	6.73E-02
BIVM	1.42	2.90E-03	7.64E-02	CFD	-2.09	6.60E-03	9.78E-02
FAM92A	1.42	4.50E-03	8.76E-02	KIAA0930	-2.1	0.00E+00	4.06E-02
TTC8	1.42	7.60E-03	1.03E-01	IQSEC1	-2.1	2.00E-04	4.42E-02
NET1	1.41	1.00E-04	4.06E-02	PLXNB2	-2.11	1.40E-03	6.48E-02
STRADB	1.41	2.00E-04	4.42E-02	HCST	-2.11	2.70E-03	7.38E-02
PRKAR2B	1.41	3.00E-04	4.98E-02	PITPNM1	-2.11	8.40E-03	1.07E-01
ZNF33B	1.41	8.00E-04	5.85E-02	HLA.DMB	-2.12	1.00E-03	6.09E-02
LRRCC1	1.4	3.90E-03	8.43E-02	S100A6	-2.12	7.60E-03	1.03E-01
PEX13	1.4	9.80E-03	1.11E-01	SEMA4A	-2.14	4.00E-04	5.14E-02
FAIM	1.39	7.70E-03	1.03E-01	FCGRT	-2.14	8.00E-04	5.85E-02
RICTOR	1.39	8.90E-03	1.08E-01	IRF7	-2.14	4.30E-03	8.70E-02
DDHD2	1.38	1.00E-04	4.42E-02	ITPRIP	-2.15	8.00E-04	5.85E-02
CCNG1	1.38	6.50E-03	9.78E-02	GBP2	-2.15	9.70E-03	1.11E-01
ORC4	1.37	2.00E-04	4.61E-02	TSPO	-2.16	2.00E-04	4.42E-02
MNAT1	1.37	1.00E-03	6.23E-02	CAPG	-2.16	1.20E-03	6.43E-02
IFT74	1.37	1.50E-03	6.62E-02	MAST3	-2.16	3.20E-03	7.79E-02
ZNF664	1.37	3.10E-03	7.79E-02	CCDC88B	-2.16	8.10E-03	1.05E-01
ABCD3	1.37	5.60E-03	9.23E-02	GLIPR2	-2.17	6.30E-03	9.75E-02
GCFC2	1.36	1.90E-03	6.76E-02	CDKN2D	-2.18	4.50E-03	8.76E-02
PRKD3	1.36	2.40E-03	7.18E-02	**VAV2**	-2.19	2.40E-03	7.18E-02
TOX	1.36	4.50E-03	8.74E-02	CDC42EP2	-2.22	1.70E-03	6.73E-02
PPIP5K1	1.36	8.00E-03	1.05E-01	RXRA	-2.23	1.00E-03	6.16E-02
LANCL1	1.36	1.10E-02	1.16E-01	NFKBIZ	-2.23	3.10E-03	7.79E-02
NT5C3A	1.35	5.00E-04	5.74E-02	CTSB	-2.24	4.00E-04	5.16E-02
MMS22L	1.35	3.40E-03	7.92E-02	CXCR4	-2.24	3.30E-03	7.86E-02

FER	1.35	6.40E-03	9.78E-02	AGTRAP	-2.26	5.00E-04	5.64E-02
APOOL	1.35	6.90E-03	1.00E-01	KLF6	-2.26	2.50E-03	7.27E-02
NEDD4L	1.35	7.80E-03	1.04E-01	IL6R	-2.27	1.60E-03	6.73E-02
ZRANB2	1.35	8.10E-03	1.05E-01	TAGAP	-2.27	8.30E-03	1.06E-01
NLK	1.34	1.00E-04	4.06E-02	TRPM2	-2.28	9.00E-04	5.88E-02
SCAI	1.34	1.70E-03	6.73E-02	DDIT4	-2.28	7.00E-03	1.01E-01
SMAD5	1.34	8.80E-03	1.08E-01	LSP1	-2.29	1.00E-03	6.09E-02
ATP6V0A2	1.33	1.00E-04	4.42E-02	CD72	-2.29	1.30E-03	6.43E-02
SLC35A3	1.33	4.00E-04	5.14E-02	SELPLG	-2.3	1.40E-03	6.48E-02
CBR4	1.33	1.01E-02	1.12E-01	IER2	-2.31	5.40E-03	9.11E-02
TTC37	1.31	3.80E-03	8.28E-02	TCIRG1	-2.32	2.40E-03	7.14E-02
AMT	1.31	4.80E-03	8.88E-02	SLC43A2	-2.36	6.50E-03	9.78E-02
KLHDC2	1.3	7.00E-04	5.85E-02	PRKCD	-2.37	3.00E-04	4.61E-02
RC3H2	1.3	1.50E-03	6.62E-02	CTSD	-2.38	1.20E-03	6.43E-02
EXOC6	1.29	7.00E-04	5.85E-02	IRS2	-2.38	2.00E-03	6.85E-02
ATP2C1	1.29	1.20E-03	6.43E-02	CTSZ	-2.38	3.70E-03	8.24E-02
HBS1L	1.28	4.00E-04	5.14E-02	IFI27L2	-2.4	1.70E-03	6.73E-02
ICA1	1.28	5.00E-04	5.25E-02	P2RX7	-2.4	5.30E-03	9.10E-02
HNRNPLL	1.28	8.00E-04	5.85E-02	C1orf162	-2.4	6.50E-03	9.78E-02
SLC39A8	1.28	4.00E-03	8.47E-02	PSAP	-2.41	1.50E-03	6.65E-02
SCD	1.28	8.90E-03	1.08E-01	MILR1	-2.42	2.60E-03	7.32E-02
DZIP3	1.27	1.50E-03	6.62E-02	COTL1	-2.42	4.30E-03	8.70E-02
COX11	1.27	1.80E-03	6.73E-02	CD79A	-2.43	4.10E-03	8.57E-02
TRAPPC6B	1.27	4.90E-03	8.88E-02	MYO1F	-2.44	4.00E-04	5.16E-02
PPM1A	1.26	4.30E-03	8.70E-02	CYSTM1	-2.45	6.40E-03	9.78E-02
ZFAND1	1.26	5.60E-03	9.23E-02	IRF8	-2.46	3.40E-03	7.92E-02
MAP4K5	1.26	5.70E-03	9.31E-02	HRH2	-2.46	7.00E-03	1.01E-01
HLCS	1.26	9.10E-03	1.09E-01	DUSP5	-2.47	8.00E-03	1.05E-01
PPIL4	1.25	1.20E-03	6.43E-02	CYTH4	-2.49	2.10E-03	6.85E-02
ZNF22	1.25	2.00E-03	6.85E-02	HLA.F	-2.49	2.50E-03	7.24E-02
KIFAP3	1.25	5.30E-03	9.11E-02	ARL4C	-2.49	4.40E-03	8.71E-02
PUS7L	1.25	8.30E-03	1.06E-01	PSTPIP1	-2.49	7.10E-03	1.01E-01
PKIG	1.25	9.50E-03	1.10E-01	HIVEP3	-2.49	8.70E-03	1.08E-01
DOCK7	1.24	4.60E-03	8.76E-02	JUNB	-2.49	9.10E-03	1.09E-01
CCND2	1.24	7.10E-03	1.01E-01	UNC93B1	-2.5	4.00E-04	5.14E-02
LRPPRC	1.23	2.00E-04	4.42E-02	B3GALT4	-2.51	5.90E-03	9.44E-02
DHX40	1.22	8.00E-04	5.85E-02	ASGR1	-2.51	1.07E-02	1.15E-01
C11orf1	1.22	4.10E-03	8.50E-02	FYB	-2.52	6.30E-03	9.76E-02
TOP2B	1.22	7.50E-03	1.03E-01	NKG7	-2.52	8.40E-03	1.06E-01
PFDN4	1.22	9.50E-03	1.10E-01	RNASE6	-2.54	4.00E-03	8.43E-02
STAT5A	1.21	6.00E-04	5.80E-02	ADAP1	-2.54	9.20E-03	1.09E-01
ABCE1	1.21	1.30E-03	6.43E-02	ITGAL	-2.55	1.00E-04	4.42E-02
CAPRIN1	1.21	2.40E-03	7.14E-02	AHNAK	-2.55	1.00E-03	6.16E-02
TTC21B	1.21	9.50E-03	1.10E-01	FOS	-2.57	5.70E-03	9.34E-02
AK3	1.2	9.00E-04	6.02E-02	GRK5	-2.63	3.10E-03	7.79E-02
CDC7	1.2	1.80E-03	6.73E-02	ANXA2	-2.66	1.90E-03	6.78E-02
RAVER2	1.2	1.90E-03	6.76E-02	BTG2	-2.67	1.10E-03	6.38E-02
CGGBP1	1.2	2.00E-03	6.84E-02	SLC16A3	-2.67	3.10E-03	7.79E-02
HAUS6	1.19	3.10E-03	7.79E-02	RAB31	-2.7	1.09E-02	1.16E-01
TFDP2	1.19	4.70E-03	8.78E-02	JUN	-2.71	3.60E-03	8.18E-02
TIA1	1.19	6.90E-03	1.00E-01	NINJ1	-2.71	4.50E-03	8.76E-02
TRIM58	1.19	1.10E-02	1.17E-01	LGALS1	-2.72	1.00E-03	6.16E-02
PM20D2	1.18	1.10E-02	1.17E-01	CTSS	-2.72	9.20E-03	1.09E-01
XPOT	1.17	5.00E-04	5.64E-02	CD22	-2.72	9.90E-03	1.11E-01

ZFAND6	1.16	4.70E-03	8.78E-02	RHOB	-2.77	3.90E-03	8.43E-02
CCNB1IP1	1.16	1.03E-02	1.12E-01	LILRA2	-2.77	5.40E-03	9.17E-02
KCTD3	1.15	2.40E-03	7.14E-02	NAGK	-2.78	3.00E-04	5.12E-02
OPA1	1.15	5.70E-03	9.31E-02	ITGB2	-2.78	5.00E-04	5.64E-02
TAF2	1.15	1.09E-02	1.16E-01	IL17RA	-2.81	4.00E-04	5.14E-02
BEX4	1.14	6.00E-04	5.80E-02	CST3	-2.82	2.00E-04	4.42E-02
TIPRL	1.14	7.00E-04	5.85E-02	DUSP1	-2.84	1.70E-03	6.73E-02
CCNC	1.14	3.20E-03	7.79E-02	TNFAIP2	-2.84	1.07E-02	1.15E-01
GTF3C3	1.14	5.10E-03	9.01E-02	NFKBIA	-2.85	7.70E-03	1.04E-01
IFT88	1.14	5.10E-03	9.01E-02	DUSP2	-2.85	7.90E-03	1.05E-01
WDR48	1.13	6.00E-04	5.80E-02	CKAP4	-2.86	8.80E-03	1.08E-01
KLHL5	1.13	1.70E-03	6.73E-02	SH3TC1	-2.89	6.00E-04	5.80E-02
PIK3R1	1.13	4.60E-03	8.78E-02	MSRB1	-2.93	1.90E-03	6.78E-02
NME7	1.13	6.60E-03	9.78E-02	CSF1R	-2.93	3.10E-03	7.79E-02
IPO5	1.12	1.70E-03	6.73E-02	GRN	-2.96	2.00E-04	4.61E-02
BRWD1	1.12	1.70E-03	6.73E-02	CEBPB	-2.96	1.50E-03	6.65E-02
CEP57	1.12	2.30E-03	7.13E-02	PLK3	-2.96	1.90E-03	6.76E-02
NPM1	1.12	3.40E-03	7.92E-02	LTB	-2.99	2.50E-03	7.19E-02
LMAN1	1.12	4.30E-03	8.70E-02	ST14	-2.99	1.06E-02	1.15E-01
MTMR2	1.12	8.30E-03	1.06E-01	IGF2R	-3.01	1.10E-03	6.33E-02
PDCD4	1.11	2.00E-04	4.61E-02	CECR1	-3.02	2.00E-03	6.85E-02
OXCT1	1.11	5.20E-03	9.10E-02	RGS2	-3.02	4.40E-03	8.73E-02
SEPT11	1.11	6.50E-03	9.78E-02	RHBDF2	-3.04	4.00E-04	5.14E-02
KRR1	1.11	6.70E-03	9.88E-02	NLRP3	-3.06	6.80E-03	9.92E-02
IREB2	1.1	6.00E-04	5.80E-02	SAMHD1	-3.08	1.60E-03	6.66E-02
NUDT4	1.1	3.30E-03	7.86E-02	HVCN1	-3.08	3.30E-03	7.86E-02
TRIM24	1.1	4.10E-03	8.50E-02	APOBR	-3.13	1.30E-03	6.43E-02
SLC30A9	1.1	4.30E-03	8.70E-02	CFP	-3.15	3.00E-03	7.76E-02
TPM1	1.1	4.40E-03	8.70E-02	POU2F2	-3.26	1.30E-03	6.48E-02
RBMXL1	1.09	1.20E-03	6.43E-02	BCL6	-3.28	8.30E-03	1.06E-01
CDC27	1.09	2.30E-03	7.02E-02	ID2	-3.29	3.30E-03	7.86E-02
TBL1XR1	1.09	3.20E-03	7.79E-02	TNFAIP3	-3.29	4.30E-03	8.70E-02
MBOAT2	1.08	2.70E-03	7.38E-02	PLAUR	-3.38	7.30E-03	1.01E-01
TOMM70	1.08	2.90E-03	7.64E-02	ZFP36	-3.4	1.40E-03	6.48E-02
ABI2	1.07	3.70E-03	8.18E-02	S100A11	-3.42	5.60E-03	9.23E-02
HIBCH	1.07	5.40E-03	9.11E-02	KLF4	-3.42	6.40E-03	9.78E-02
DLD	1.07	5.70E-03	9.29E-02	C10orf54	-3.46	3.00E-04	5.14E-02
HS2ST1	1.07	9.50E-03	1.10E-01	JAML	-3.51	2.10E-03	6.85E-02
RABL3	1.07	9.60E-03	1.10E-01	TYROBP	-3.56	6.50E-03	9.78E-02
TIMM10B	1.06	5.50E-03	9.21E-02	EGR1	-3.59	3.10E-03	7.79E-02
SNRNP48	1.06	5.70E-03	9.33E-02	SLC2A6	-3.63	3.30E-03	7.86E-02
RBM7	1.06	6.60E-03	9.78E-02	CEBPD	-3.82	8.00E-04	5.85E-02
SMIM8	1.06	8.10E-03	1.05E-01	TNFRSF1B	-3.9	2.00E-04	4.61E-02
FUBP1	1.05	1.20E-03	6.43E-02	LILRB2	-3.91	7.40E-03	1.01E-01
RMND5A	1.05	2.90E-03	7.64E-02	KLF2	-4.06	2.00E-04	4.61E-02
USP33	1.05	4.30E-03	8.70E-02	LILRB4	-4.06	6.20E-03	9.72E-02
CCDC58	1.05	5.60E-03	9.26E-02	LYZ	-4.08	2.70E-03	7.40E-02
FAM98B	1.05	9.30E-03	1.10E-01	HCK	-4.08	5.10E-03	9.01E-02
TTC19	1.04	1.00E-04	4.16E-02	FAM20C	-4.12	2.00E-03	6.85E-02
RAD17	1.04	3.40E-03	7.92E-02	TYMP	-4.16	6.90E-03	1.00E-01
TNPO1	1.03	1.70E-03	6.73E-02	**CYBB**	-4.42	7.70E-03	1.03E-01
DDX1	1.03	4.30E-03	8.70E-02	FGR	-4.47	1.08E-02	1.16E-01
UBLCP1	1.03	9.50E-03	1.10E-01	CTSH	-4.51	4.40E-03	8.70E-02
TYW3	1.02	1.80E-03	6.76E-02	**NCF1**	-4.57	7.60E-03	1.03E-01

CHD1L	1.02	3.30E-03	7.86E-02	MPEG1	-4.65	5.90E-03	9.41E-02
NUDT21	1.02	3.90E-03	8.43E-02	PLEC	-4.75	1.00E-04	4.42E-02
COQ8A	1.02	5.20E-03	9.08E-02	FCN1	-5.95	2.50E-03	7.27E-02
ZC3H8	1.02	9.10E-03	1.09E-01	LRP1	-6.18	4.70E-03	8.78E-02
HIGD1A	1.02	9.70E-03	1.10E-01	S100A9	-6.95	4.00E-04	5.14E-02
CTPS2	1.01	2.00E-03	6.84E-02	**S100A8**	-7.1	1.20E-03	6.43E-02
ZNF639	1.01	7.00E-03	1.00E-01				
CFAP36	1.01	7.00E-03	1.01E-01				
IP6K2	1.01	1.02E-02	1.12E-01				

Copyright: © 2021 by the authors. Licensee MDPI, Basel, Switzerland. This article is an open access article distributed under the terms and conditions of the Creative Commons Attribution (CC BY) license (http://creativecommons.org/licenses/by/4.0/).
